# *In silico* Analysis Suggests Common Appearance of scaRNAs in Type II Systems and Their Association With Bacterial Virulence

**DOI:** 10.3389/fgene.2018.00474

**Published:** 2018-10-17

**Authors:** Jelena Guzina, Wei-Hua Chen, Tamara Stankovic, Magdalena Djordjevic, Evgeny Zdobnov, Marko Djordjevic

**Affiliations:** ^1^Institute of Physiology and Biochemistry, Faculty of Biology, University of Belgrade, Belgrade, Serbia; ^2^Multidisciplinary PhD Program in Biophysics, University of Belgrade, Belgrade, Serbia; ^3^Swiss Institute of Bioinformatics and Department of Genetic Medicine and Development, University of Geneva, Geneva, Switzerland; ^4^Institute of Physics Belgrade, University of Belgrade, Belgrade, Serbia

**Keywords:** CRISPR/Cas, small RNA, scaRNA, tracrRNA, bacterial pathogenicity, non-canonical CRISPR/Cas functions

## Abstract

In addition to its well-established defense function, CRISPR/Cas can also exhibit crucial non-canonical activity through endogenous gene expression regulation, which was found to mainly affect bacterial virulence. These non-canonical functions depend on scaRNA, which is a small RNA encoded outside of CRISPR array, that is typically flanked by a transcription start site (TSS) and a terminator, and is in part complementary to another small CRISPR/Cas-associated RNA (tracrRNAs). Identification of scaRNAs is however largely complicated by the scarcity of RNA-Seq data across different bacteria, so that they were identified only in a relatively rare CRISPR/Cas subtype (IIB), and the possibility of finding them in other Type II systems is currently unclear. This study presents the first effort toward systematic detection of small CRISPR/Cas-associated regulatory RNAs, where obtained predictions can guide future experiments. The core of our approach is *ab initio* detection of small RNAs from bacterial genome, which is based on jointly predicting transcription signals – TSS and terminators – and homology to CRISPR array repeat. Particularly, we employ our improved approach for detecting bacterial TSS, since accurate TSS detection is the main limiting factor for accurate small RNA prediction. We also explore how our predictions match to available RNA-Seq data and analyze their conservation across related bacterial species. In Type IIB systems, our predictions are consistent with experimental data, and we systematically identify scaRNAs throughout this subtype. Furthermore, we identify scaRNA:tracrRNA pairs in a number of IIA/IIC systems, where the appearance of scaRNAs co-occurs with the strains being pathogenic. RNA-Seq and conservation analysis show that our method is well suited for predicting CRISPR/Cas-associated small RNAs. We also find possible existence of a modified mechanism of CRISPR-associated small RNA action, which, interestingly, closely resembles the setup employed in biotechnological applications. Overall, our findings indicate that scaRNA:tracrRNA pairs are present in all subtypes of Type II systems, and point to an underlying connection with bacterial virulence. In addition to formulating these hypotheses, careful manual curation that we performed, makes an important first step toward fully automated predictor of CRISPR/Cas-associated small RNAs, which will allow their large scale analysis across diverse bacterial genomes.

## Introduction

CRISPR/Cas is an adaptive prokaryotic immune system that protects the cell against invading nucleic acids (such as phage or plasmid), through a concerted action of its two components – CRISPR array and CRISPR associated (Cas) proteins (Makarova et al., 2006; Barrangou et al., 2007). CRISPR array consists of a (variable) number of short repetitive elements, each followed by unique spacer sequence (Bolotin et al., 2005). These spacer sequences, along with flanking tandem repeat, give rise to a guide RNA molecule (called crRNA), through which specific targets are recognized and, eventually, cleaved (Brouns et al., 2008). This target cleavage, as well as prior steps of spacer acquisition and CRISPR transcript processing, are aided by the (mainly nucleolytic) activity of Cas proteins.

CRISPR/Cas systems are divided into six main Types (I-VI) and a number of subtypes, according to a specific combination of effector Cas proteins and the adjacent CRISPR array structure (Makarova et al., 2011; Jiang and Doudna, 2015). Type I and III systems, encode for multimeric Cas protein complexes. In distinction, Type II systems, which are often found across the genomes of pathogenic bacteria (Chylinski et al., 2014; Sampson and Weiss, 2014), employ a single Cas9 protein as effector molecule (Deltcheva et al., 2011). Type II systems are also unique, as they require two distinct non-coding RNA species. First is crRNA (a secondary product of CRISPR array expression), while the second is encoded outside the array and represents a novel CRISPR-associated small RNA. This tracrRNA (*trans* activating crRNA) has a partial complementarity with crRNA, with whom it forms a duplex, that acts as a platform for Cas9 recruitment (Jinek et al., 2012). Formation of the effector complex is followed by target recognition through base-pairing with complementary segments on crRNA, and subsequent target cleavage, mediated by Cas9 (Sternberg et al., 2014; Jiang et al., 2015; Lim et al., 2016).

As CRISPR/Cas protects the genome integrity by specifically destroying invasive genetic elements, its activity inevitably reduces the rate of horizontal gene transfer (HGT) (Marraffini and Sontheimer, 2008). However, under some circumstances, HGT can be highly beneficial to the host cell. In particular, in pathogenic bacteria, virulence factor or antibiotic resistance genes are frequently acquired through HGT (Novick and Ram, 2016; Messerer et al., 2017), which may lead to significant increase in their fitness. Consequently, it would not be surprising that pathogenic bacteria, with actively expressed CRISPR/Cas systems, explore additional avenues for enhancing their virulence/environmental adaptability, to compensate for the reduced rate of HGT.

In fact, recently discovered non-canonical functions of (predominantly Type II) CRISPR/Cas systems fit very well into this assumption (Hille and Charpentier, 2016). A growing body of evidence suggests that the alternative CRISPR/Cas functions are intrinsically related to bacterial virulence, presumably by controlling cell envelope composition (Hatoum-Aslan and Marraffini, 2014; Sampson and Weiss, 2014; Ratner et al., 2015) through regulation of endogenous gene expression. In majority of cases, however, a detailed understanding of the mechanisms by which Type II CRISPR/Cas systems affect virulence is missing. Only in a bacterium *Francisella novicida U112*, it is well established that the non-canonical activity of its Type IIB CRISPR/Cas system hinders the activation of host immunity, by down-regulating the expression of BLP – which is the elicitor of immune response, normally found in the cell envelope of *F. novicida* (Sampson et al., 2013).

The regulation of BLP expression occurs at mRNA level, through the action of guide RNA duplex, loaded with Cas9 endonuclease (Sampson et al., 2013). Interestingly, instead of the canonical crRNA, another small CRISPR-associated RNA species (called scaRNA) appears in complex with tracrRNA. As in canonically acting Type II systems, the RNA duplex (herein scaRNA:tracrRNA pair) enables target recognition, which means that repurposing of the entire CRISPR/Cas machinery onto new targets can be achieved by expressing only one additional CRISPR/Cas associated small RNA (scaRNA) molecule. This indicates that scaRNA:tracrRNA paradigm could act as a universal avenue for delivering a wide repertoire of non-canonical functions in Type II systems, through endogenous gene regulation at mRNA level.

In line with this, the crucial step in elucidating the mechanisms by which non-canonically acting Type II CRISPR/Cas systems affect endogenous gene regulation would be systematic detection of small CRISPR-associated RNAs across different bacterial genomes. In general, the most common practice for small non-coding RNA detection is through RNA-Seq; however, RNA-Seq data are still very scarce across different bacterial species and there also comes a question if conditions under which RNA-Seq is performed match those under which CRISPR/Cas associated small RNAs are active. On the other hand, *ab initio* computational search of bacterial genomes can be applied to any genome sequence, thus enabling exhaustive search of CRISPR-associated small RNAs throughout different Type II systems of virulent bacteria, which represents the main objective of this study. This study presents the first attempt to computationally predict small non-coding RNAs within (Type II) CRISPR/Cas loci, even though these molecules have been recognized as carriers of important regulatory and effector roles in both canonical and non-canonical CRISPR/Cas functioning for some time.

In general, different approaches are used to tackle the problem of small RNA detection directly from the genomic sequence, including comparative genomics-based approaches, secondary structure/thermodynamic stability prediction, and also detecting the associated transcription signals (transcription start sites – TSS and terminators) (Sridhar and Gunasekaran, 2013). Note that small non-coding RNAs in bacteria are usually deprived of characteristic secondary structure, distinguishable nucleotide statistics, and also high level of conservation across distantly related genomes, which eventually narrows the range of reliable search predictors to TSS and terminators. In addition to detecting transcription signals, *ab initio* prediction of small RNAs associated to CRISPR/Cas loci can be further aided through detection of characteristic homology with the CRISPR array, as segments of both tracrRNA and scaRNA molecules display complementarity to the forward/reverse strand of the array direct repeat (Sampson et al., 2013).

The strategy introduced above makes the outline of our search procedure, where major strength is an improved approach for TSS detection, that we previously developed, as this step was shown to be the main limiting factor for small RNA detection accuracy (Argaman et al., 2001). Namely, we showed that our weight matrix-based procedure, which implements *de novo* alignment of bacterial RpoD promoter sequences and also accounts for specificity of sequences flanking -35 and -10 elements, leads to 50% false positive reduction (Nikolic et al., 2017). Although this significantly improves the search accuracy, the false positive rate is still high, so that manual curation of the obtained results is needed. Therefore, as true predictions for small CRISPR-associated RNAs, we consider cases when segments homologous to array repeat are clearly bound with transcription signals (TSS and terminators), while discarding the remaining hits. Also, as secondary evidence to these *ab initio* predictions, we use conservation analysis of the predicted small RNA units in related bacterial species, and RNA-Seq data mining, where available.

The analysis presented here focuses on virulent bacterial strains that harbor different Type II CRISPR/Cas systems. As subtype IIB is currently the only one with experimental evidence of scaRNA presence, members of this subtype – the experimentally analyzed system of *F. novicida U112* and two additional systems (from *Legionella pneumophila 130b* and *Wolinella succinogenes DSM 1740*) that share equivalent CRISPR/Cas locus architecture will be used to parameterize the search procedure. Potential ubiquity of scaRNA:tracrRNA paradigm will be assessed through the analysis of different Type IIA and IIC systems, where in particular we will incorporate all the examples from literature, where the connection between CRISPR/Cas components and virulence was experimentally indicated (Louwen et al., 2013; Sampson et al., 2013; Gunderson et al., 2015). Namely, in such systems, it might be expected that CRISPR/Cas involvement in virulence is mediated by small CRISPR-associated RNAs, analogously to what was observed in *F. novicida U112*. Finally, in addition to pathogenic, we will also analyze a number of non-virulent strains, to further test the virulence-related role of small CRISPR-associated RNA molecules.

Consequently, the study presented here is a first step toward a systematic search of CRISPR/Cas associated small RNAs. Note that such systematic search is a complicated problem, where appropriate transcription signals must be found on appropriate distance from each other, must contain regions of homology with array repeats, and must be in an appropriate orientation with respect to each other and CRISPR array repeat. In addition, one also has to take care that, due to limited accuracy of transcription signal predictions, and a possibility of different mechanism of transcription initiation/termination in some bacterial strains, some of the predicted transcription signals might be missing, while all other signals are in place. Due to this, we here present manually curated predictions, for a number of CRISPR/Cas loci, whose selection was aided by careful literature search. Where possible, these predictions are compared with available RNA-Seq data and subjected to conservation analysis. The goal is to obtain a set of high confidence (manually curated) predictions, which can be used to form rational hypothesis on the role of CRISPR/Cas associated small RNAs through Type II systems, on possible alternative mechanisms of their action, and their involvement in bacterial virulence. Equally important, this high-confidence set provides a starting point for future experiments (where otherwise significant resources might be wasted), and a training set for future automated CRISPR/Cas associated small RNA prediction tools.

## Methods

### Bacterial Strain Selection

The analyzed strains were gathered from the **Supplementary Table S1**, given in Chylinski et al. (2014), which provides a list of bacterial strains harboring *cas9* – a signature gene for Type II CRISPR/Cas systems. Among these, we focused only on pathogenic strains, where the presence of additional *cas* genes (normally found in Type II CRISPR/Cas systems) was also indicated. This was to enable distinguishing between (putatively) complete CRISPR/Cas loci, needed for further analysis, and orphan (stand-alone) *cas9* genes, that also appear across bacterial genomes. Note, however, that the presence of the entire set of Type II-associated genes (*cas1, cas2*, and subtype-specific *cas4* and *csn2*) was not considered obligatory.

We selected in total 12 bacterial strains (**Table 1**), that cover all three subtypes (IIA, IIB and IIC), including literature examples, where CRISPR/Cas was implicated in bacterial virulence. As our search assumes the presence of complete CRISPR/Cas loci, the genomic sequences corresponding to the above strains (gathered from the GenBank) were next inspected for the presence of annotated CRISPR arrays, in the vicinity of *cas9* genes. When this annotation was not available, the genomic sequences were submitted to CRISPRFinder (Grissa et al., 2007), instead.

**Table 1:** Information about the Type II CRISPR/Cas components of analyzed bacterial strains.

Bacterial strain	GI number						DR sequence (5′ – 3′)
	genome/ scaffold	Cas9	Cas1	Cas2	Cas4	Csn2	
**Type IIA**		
*Listeria innocua clip1 11262*	16799079	499300419	489869401			908637547	GTTTTGTTAGCATTCAAA ATAACATAGCTCTAAAAC
*Mycoplasma gallisepticum F*	385325853	504387687	504387688	504387689		504387690	GTTTTAGCACTGTACAAT ACTTGTGTAAGCAATAAC
*Streptococcus pyogenes M1GAS*	602625715	13622193	13622194	13622195		13622196	GTTTTAGAGCTATGCTGT TTTGAATGGTCCCAAAAC
*Streptococcus mutans UA159*	347750429	24379809	24379808	24379807		24379806	GTTTTGGAACCATTCGAA ACAACACAGCTCTAAAAC
*Lactobacillus salivarius UCC118*	90820184	90820277	90820280	90820281		90820282	GTTTCAGAAGTATGTTAAA TCAATAAGGTTAAGACC
*Listeria monocytogenes SLCC 2482*	404285367	489827017	489827018	489819839		489827019	GTTTTGGTAGCATTCAAAA TAACATAGCTCTAAAAC
**Type IIB**		
*Francisella novicida GA99-3548*	148535189	151571895			151571896		CTAACAGTAGTTTACCAAA TAATTCAGCAACTGAAAC
*Francisella novicida U112*	118496615	489129153	489123804	489116840	500053719		CTAACAGTAGTTTACCAAA TAATTCAGCAACTGAAAC
*Legionella pneumophila 130b*	307608751	307608922	307608923	307608924	307608925		CCAATAATCCCTCATCTAAA AATCCAACCACTGAAAC
*Wolinella succinogenes DSM 1740*	34556458	499451967	499451968	499451969	1174233214		GCAACACTTTATAGCAAATC CGCTTAGCCTGTGAAAC
**Type IIC**		
*Neisseria lactamica 020 06*	313667359	503214802	489807126	488143358			ATTGTAGCACTGCGAAATG AGAAAGGGAGCTACAAC
*Neisseria meningitidis ATCC 13091*	305682232	488143352	488143355	488143358			ATTGTAGCACTGCGAAATG AGAAAGGGAGCTACAAC
*Campylobacter jejuni 81116*	157414322	157386708	157386707	157386706			GTTTTAGTCCCTTTTTAAAT TTCTTTATGGTAAAAT
*Pasteurella multocida PM70*	15601865	499209493	492115307	499209491			GTTGTAGTTCCCTCTCTCAT TTCGCAGTGCTACAAT
*Haemophilus parainfluenzae T3T1*	345428590	503831578	754507616	503831580			ATTATAGCACTGCGAAATGA AAAAGGGAGCTACAAC


Note that, for a number of selected strains, the corresponding genomes were deposited in GenBank in the form of genomic scaffolds. In such cases, the genomic scaffolds were subjected to BLAST (tblastn) search (Altschul et al., 1990), against the Cas9 protein belonging to the same strain, to identify the scaffold harboring Type II CRISPR/Cas system. The procedure for detecting CRISPR array within such a scaffold corresponds to the procedure described above.

In addition to bacterial strains noted above, to parameterize the search procedure, we also analyzed three Type IIB strains with CRISPR/Cas organization equivalent to the one in which scaRNA was experimentally inferred.

### Intergenic Region and CRISPR Loci Extraction

The search for small CRISPR-associated RNAs was limited to intergenic regions within CRISPR/Cas locus. Note that CRISPR locus includes CRISPR array, *cas* genes, intergenic regions between them, and two additional intergenic regions, that flank *cas* genes and CRISPR array. Intergenic regions of minimum 50 bp length were searched, in accordance with typical length of transcription signals (core promoters and terminators), to be detected in the search. Both strands of the intergenic regions (forward and reverse) were searched.

These intergenic regions were searched for regions of homology to the array direct repeats, and to detect TSS (core promoters). Additionally, we defined another set of regions for terminator search, by extending the existing regions for 15 bp in the downstream direction; the extension corresponds to the length of the segment that is used for U-score calculation, i.e., to search segment in the terminator search, see Section “Terminator Prediction.”

### Small RNA Search

The intergenic regions were first queried for homology to the array direct repeat (oriented by default on the forward genomic strand). The regions where homology could successfully be inferred were next submitted to promoter and terminator search in both orientations (forward and reverse), as the transcription orientation of the array itself (and, therefore, correct annotation of the direct repeat) is not known in advance. To infer the array orientation, the intergenic regions upstream and downstream from the CRISPR array were searched for TSS and terminators; note that information on the array orientation is necessary for functional annotation of predicted small RNAs. Namely, tracrRNAs should harbor segments complementary to the array direct repeats; therefore, the prediction of the segment homologous to repeat, which is transcribed in the reverse orientation, with respect to the array, corresponds to putative tracrRNA. Analogously, the prediction of the segment homologous to repeat, transcribed in the same orientation as the array itself, determines putative scaRNA.

#### Predicting the Homology With the Array Direct Repeat

The extracted intergenic regions were queried for homology to the array direct repeats by using local pair-wise sequence alignment. As suboptimal alignments are also reported, this enables prediction of more than one small RNA unit per intergenic region. Note that the reported alignment scores take into account variations in the alignment length, which enabled setting a uniform threshold for all the reported hits, regardless of their length. This threshold was set according to the lowest score reported for the hits associated with the inferred small RNAs in *F. novicida U112*, *W. succinogenes DSM 1740* and *L. pneumophila 130b* Type II systems. In the case when only one (or none) of the reported alignments exceeds the established threshold, the next highest-scoring hit was considered as positive prediction, if the associated score is not smaller by more than 20% with respect to the threshold.

#### TSS Predictions

The core (TSS) predictions, relied on the supervised, weight matrix-based search, that uses *de novo* alignment of RpoD promoter elements, corresponding to experimentally inferred TSS (322 sequences from RegulonDB database (Huerta et al., 1998; Djordjevic, 2011). The weight matrices were constructed for -35, -10 extended (i.e., -15) and short -10 element, also weights corresponding to different spacer lengths in the alignment were used; for details on the weight matrix construction see e.g., (Djordjevic, 2011). Analogously to searching segments that are homologous to array repeat (see Predicting the Homology With the Array Direct Repeat), the search threshold was set to the value that enables reproducing small RNA units in *F. novicida U112*, *W. succinogenes DSM 1740* and *L. pneumophila 130b* strains.

#### Terminator Prediction

The terminator search follows the established concept for recognizing Rho-independent terminators (Ermolaeva et al., 2000), which is based on detecting experimentally observed features of Rho-independent *Escherichia coli* terminators: the free energy of the terminator stem-loop structure, the stem and loop size, the number of GC-pairs in the stem, and also the U-enrichment score.

Intergenic regions were checked for the presence of appropriate stem-loop structures within sequence frames spanning from 11 to 50 bp, which correspond, respectively, to the minimal and maximal length of the stem-loops found in experimentally inferred *E. coli* terminators (Ermolaeva et al., 2000). The stem-loops were predicted by calculating the minimum free energy (ΔG) associated with the secondary sequence structure, using a thermodynamic nearest-neighbor approach. The ΔG cut-off was set to -6 kcal/mol, the value that enabled reproduction of the inferred small RNA units in *F. novicida U112*, *W. succinogenes DSM 1740* and *L. pneumophila 130b* strains. In addition to this, the predicted stem-loop structures were constrained with the request of having at least 3 GC pairs in the stem, which should be at least 4 bp long, also corresponding to the observed features of *E. coli* terminators.

Besides appropriate stem-loop, another feature that characterizes (*E. coli*) Rho-independent terminators is a U-rich downstream segment. In line with this, U-score was calculated for every stem-loop structure within flanking 15 bp-long segment, according to the empirical equation from Ermolaeva et al. (2000), where the search threshold was set to -2.3, according to the same criteria as above. Note here that the U-score threshold was liberalized with respect to the value characteristic of predictions associated with experimentally inferred small RNAs in the training set (Type II system of *F. novicida U112*), as this feature is most pronounced in so called L-shaped terminators, that are widely present in the genome of *E. coli*. Namely, the genomes of other bacteria are often enriched with different types of Rho-independent terminators (Mitra et al., 2009; Peters et al., 2011), that could be omitted in the search characterized by a very restrictive *U*-score threshold.

Also, note that, in the case of predicting overlapping terminators, the search reports only the hit associated with a better ΔG score, and also that among the predicted terminators, only the ones that (putatively) act as downstream boundaries of the small RNA expression units/CRISPR array are considered as positive predictions (and, therefore, shown in the corresponding transcription schemes).

#### Conservation Analysis

The predicted small RNAs were used as queries for the search against the NCBI non-redundant nucleotide sequence database (nt, downloaded in July 2016) using BLASTN (version 2.3.0+). An arbitrary *E*-value cutoff of 1e-4 was used to filter the BLAST hits. If a small RNA had BLAST hits above the threshold, the species information was obtained for each of the BLAST hits from NCBI, then an in-house PERL script was used to calculate the last common ancestor (LCA) of all the species on the NCBI taxonomic tree, including the species where the small RNA was detected. Multiple sequence alignments and conservation profiles shown in **Supplementary Figure S1** are generated by BLAST and ClustalX (Larkin et al., 2007).

#### Expression Analysis

To find whether the predicted small RNAs are expressed, for each species of interest, all available RNA-Seq datasets (if available) were obtained from the NCBI SRA database. A BLASTN (Zhang et al., 2000) search (version 2.3.0+) was then performed by using the small RNAs of the same species as queries. An arbitrary *E*-value cutoff of 1e-4 was used to filter the BLAST hits; in addition, the sequence identity of an aligned region should be >95%.

## Results

Our main hypothesis is that small CRISPR-associated RNAs act as common mediators in non-canonical functions of Type II CRISPR/Cas systems; note that CRISPR/Cas components have been repeatedly linked to host-pathogen interactions and bacterial virulence, though exact mechanism for such link was not established except in one case (Sampson and Weiss, 2014; Ratner et al., 2015). To assess the ubiquity of these RNA species across all three Type II subtypes in pathogenic bacteria, we employ a computational procedure based on detecting TSS and terminators, as these signals specify both the length and orientation of putative transcription units.

As outlined in the Introduction (see also Methods), the search of small CRISPR-associated RNAs also includes probing the predicted transcription units for homology with the array tandem repeat, where putative tracrRNAs should exhibit homology with complementary, and scaRNAs with direct repeat strand, according to their established roles in Type IIB system of *F. novicida U112* (Sampson et al., 2013). Therefore, an important step in our search – that allows distinguishing tracrRNA from scaRNA species – is predicting transcription orientation of the CRISPR array. Note here that in Type II systems transcription orientation of the array does not necessarily coincide with the orientation of *cas* genes (Zhang et al., 2013), yet is also unambiguously defined by the layout of accompanying transcription signals; hence, it will be inferred in the same way as for small RNA expression units.

Currently available information on CRISPR-associated small RNAs suggests close proximity to either CRISPR array, as seen in the Type IIB system of *F. novicida U112* (Sampson et al., 2013), or *cas* genes, as seen for tracrRNAs across diverse Type IIA/IIC systems (Chylinski et al., 2014); therefore, we search only those intergenic regions found within CRISPR/Cas loci. To corroborate our search procedure, and to start testing our hypothesis of ubiquity of scaRNA:tracrRNA pairs, we first concentrate on subtype IIB members, that share equivalent locus architecture, as these systems enable us: (i) to validate obtained predictions against the existing experimental evidence for tracrRNA and scaRNA molecules (available for *F. novicida U112*); (ii) to examine if in Type IIB-harboring species other than *F. novicida* (precisely, *L. pneumophila 130b* and *W. succinogenes DSM 1740*) we obtain equivalent predictions for scaRNAs, which would preliminary test the hypothesis, i.e., indicate that scaRNA:tracrRNAs are ubiquitous in at least this Type II CRISPR/Cas subtype. Moreover, note that in *L. pneumophila 130b*, the connection between CRISPR/Cas components and virulence was experimentally established (though not the exact mechanism by which this is achieved), so the appearance of scaRNA would be highly anticipated in this Type IIB system (Gunderson et al., 2015).

### scaRNA:tracrRNA Pairs Appear Common in Type IIB Systems

**FIGURE 1:**
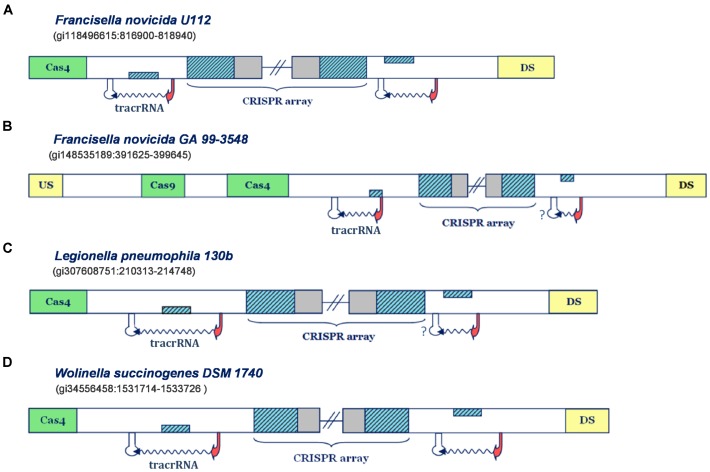
Predictions of small CRISPR/Cas-associated RNAs in Type IIB CRISPR/Cas systems. The organization of Type IIB systems found in *Francisella novicida U112*, *Francisella novicida GA 99-3548*, *Legionella pneumophila 130b* and *Wolinella succinogenes DSM 1740* is presented in figure **(A–D)**, respectively. The CRISPR array direct repeat and segments homologous to repeat are indicated with blue diagonally-hatched boxes, CRISPR spacer sequence with gray, *cas* genes with green, and the genes flanking CRISPR/Cas locus with yellow boxes. Predicted small RNAs are specified by the upstream promoter (indicated as red arrow) and downstream terminator signals (indicated as white stem-loop structures), complemented with the wavy arrow pointing to predicted transcription direction. Note that the stem-loop structures associated with a question mark correspond to possible false negative terminator predictions. **Figures 1**–**4** schematically represent the most informative results from **Supplementary Table S1**, while for more details the reader should refer to **Supplementary Table S1**.

IIB is the least frequent subtype among Type II CRISPR/Cas systems (Chylinski et al., 2014), so the experimental information related to this subtype is also rarely available. In line with this, the information regarding scaRNA:tracrRNA paradigm is currently associated only with the system of *F. novicida U112*, which thus becomes central for parameterizing and validation of our search procedure. Note that in **Figure 1**, we also analyze Type IIB system of *F. novicida GA 99-3548*, though independently from the parameterization procedure, due to putative close resemblance to Type IIB locus of *F. novicida U112*. As noted above, in **Figure 1** we also include two additional Type IIB representatives, which share the system architecture with *F. novicida U112* (systems of *L. pneumophila 130b* and *W. succinogenes DSM 1740).*

In **Figure 1A** one can observe that the predicted transcription scenario for the Type IIB system of *F. novicida U112* aligns fully with the available experimental information (Sampson et al., 2013). Namely, the predicted transcription signals correspond to inverse orientation of all system components, so that the segment homologous to repeat, found right upstream from the array, on the direct strand, corresponds to scaRNA, while the one downstream from the array, found on the reverse strand, gives tracrRNA. Note here that in the figure panels we show only promoter and terminator predictions that flank the segments homologous to array repeat on the upstream and downstream edge, whereas the exhaustive information (including exact scores and coordinates) for all the predictions above the search thresholds, is given in the **Supplementary Table S1**.

An equivalent scenario is evident in the Type IIB system of *F. novicida GA 99-3548* (**Figure 1B**). Namely, the segments homologous to repeat in this system also appear on the direct strand, downstream from the array, and on the reverse strand, upstream to it. Both of these segments are confined within multiple (and sometimes strong) transcription signals in the “-” orientation, thus giving putative scaRNA/tracrRNA expression units. Since the array is also “-” oriented, due to presence of multiple and strong upstream promoters, the expression unit preceding the array corresponds to scaRNA, and the succeeding to tracrRNA. Note, however, that the predicted scaRNA appears to be deprived of the downstream terminator signal, which might be due to (i) scaRNA being transcribed as one long transcript with CRISPR array (and later being processed to a separate small RNA), or (ii) a missing terminator prediction, as our search predicts only L-shaped Rho-independent elements. Interestingly, the system of *F. novicida GA 99-3548* is deprived of *cas1*/*cas2* genes, which are in charge of the adaptation step (i.e., array immunization with novel spacer sequences) (Jackson et al., 2017). In fact, such loss may be consistent with non-canonical system activity mediated by scaRNA:tracrRNA complex, as the absence of Cas1/Cas2 nucleases impairs the array immunization with new spacers. Due to this, the system eventually becomes outdated in terms of its immune response, i.e., incapable of responding to concurrent phage infections, which might promote its alternative (non-canonical) functions.

We saw previously that Type IIB systems of *F. novicida* strains share equivalent organization of the transcription units, however, predictions of individual transcription signals are unrelated in these strains. Actually, the equivalent predicted organization of CRISPR loci also appears in Type IIB systems of *W. succinogenes DSM 1740* and *L. pneumophila 130b*, where one can easily observe that the layout of predicted promoters and terminators suggests the expression of all system components from the reverse strand. Therefore, transcription units located upstream from the array, where the segments homologous to repeat are located on the direct strand give scaRNA, while the downstream units, with the segments homologous to repeat, that are inversely oriented, give tracrRNA.

It is interesting to note that in *L. pneumophila 130b*, just like in *F. novicida GA 99-3548*, scaRNA and CRISPR array might be jointly transcribed, due to absence of intervening terminator signals. To some extent, this could also be the case in *F. novicida U112*, as terminators found downstream from scaRNA and CRISPR array are rather week, so that read-through transcription might occur. Additionally, in *W. succinogenes DSM 1740* putative tracrRNA appears as stand-alone expression unit, while scaRNA might also be expressed with the CRISPR array, due to relatively weak transcription termination signal (see **Supplementary Table S1**). In such a case, the appearance of strong promoter signals immediately upstream from putative tracrRNAs might enable fine-tuning expression levels, which could, in turn, be important to balance the pairing with crRNAs and scaRNAs. In other words, such regulation might enable concomitant canonical and non-canonical system functioning, when/if necessary.

To summarize, all Type IIB systems are associated with clear predictions for scaRNA, including the system of *L. pneumophila 130b*, where the connection between CRISPR/Cas components and virulence was experimentally established. Importantly, the predictions obtained in Type IIB system of *F. novicida U112* are in excellent agreement with the experimental information, thus corroborating the suitability of our search procedure.

### Presence of Only tracrRNA in Type II Systems Appears Associated With Non-virulent Strains

**FIGURE 2:**
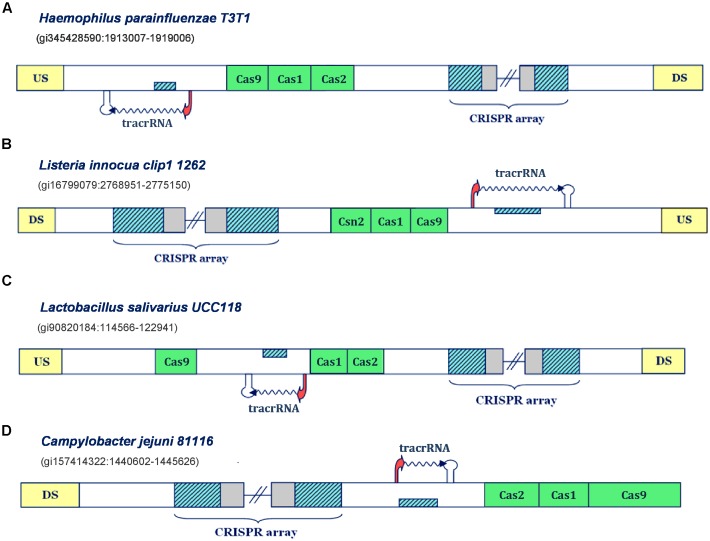
Predictions of tracrRNAs in different Type IIA and IIC CRISPR/Cas systems. The organization of Type II systems found in *Haemophilus parainfluenzae T3T1*, *Listeria innocua clip1 1262*, *Lactobacillus salivarius UCC118* and *Campylobacter jejuni 81116* is presented in figure **(A–D)**, respectively. The system components are indicated analogously as in **Figure 1**.

We next explore the transcription scenarios in Type IIC and Type IIA CRISPR/Cas systems. Appearance of scaRNA has not yet been evidenced in these subtypes, but components of these systems were implicated in non-canonical CRISPR/Cas functions. Precisely, components of Type IIC systems in *Campylobacter* and *Neisseria* species were shown to be important for promoting host attachment and intracellular replication (Louwen et al., 2013; Sampson et al., 2013) – processes vital for infection establishment. However, in distinction to Type IIB systems, where we consequently encounter scaRNA:tracrRNA pairs, in Type IIA and Type IIC systems, there are clear examples with only one putative small RNA expression unit (**Figure 2**).

Namely, in Type IIC system of *Haemophilus parainfluenzae T3T1* (**Figure 2A**), we observe one inversely-oriented segment homologous to repeat, upstream from *cas* genes, confined within strong promoter and terminator signals in the reverse orientation. Note here that the transcription orientation of the CRISPR array cannot be unambiguously defined, due to the lack of closely positioned upstream promoter signals; however, the “+” oriented transcription seems more reasonable, as it enables annotation of tracrRNA (instead of scaRNA) upstream from *cas* genes, as this molecule is indispensable for both canonical and non-canonical system functioning. Similarly, in the Type IIC system of *Listeria innocua clip11262* (**Figure 2B**), the expression of tracrRNA occurs from the only segment homologous to array repeat, which is bound with promoter and terminator signals, and is located on the direct strand of the intergenic region upstream from *cas* genes. The (only) promoter preceding the array is located on the reverse strand, so that the transcription of the segment homologous to repeat, preceding *cas* genes, produces small RNA that is complementary to the array tandem repeat.

On the other hand, in the Type IIA system of *Lactobacillus salivarius UCC118*, the segment homologous to repeat is found between *cas* genes; precisely, on the direct strand, with transcription signals in the “-” orientation. Therefore, the expression of the CRISPR array occurs from the direct strand, even though this transcription orientation lacks properly positioned promoter signals. Finally, we observe similar scenario in the Type IIC system of *Campylobacter jejuni 81116* (**Figure 2D**), where the expression of CRISPR array should also occur on the direct strand, so that the inversely oriented segment homologous to repeat, that is transcribed from the direct strand, could give rise to tracrRNA. Consequently, these findings demonstrate that our approach can identify transcription units corresponding to tracrRNA in CRISPR/Cas loci.

Notably, all the bacteria with CRISPR/Cas loci harboring one small RNA (i.e., tracrRNA, presented in **Figure 2**) are categorized as non-virulent strains, where *C. jejuni 81116* represents the only exception, which is consistent with our initial hypothesis on non-canonical CRISPR/Cas activities being related with virulence.

### Presence of scaRNA:tracrRNA Pairs in Type II Systems Appears Common and Associated With Virulent Strains

**FIGURE 3:**
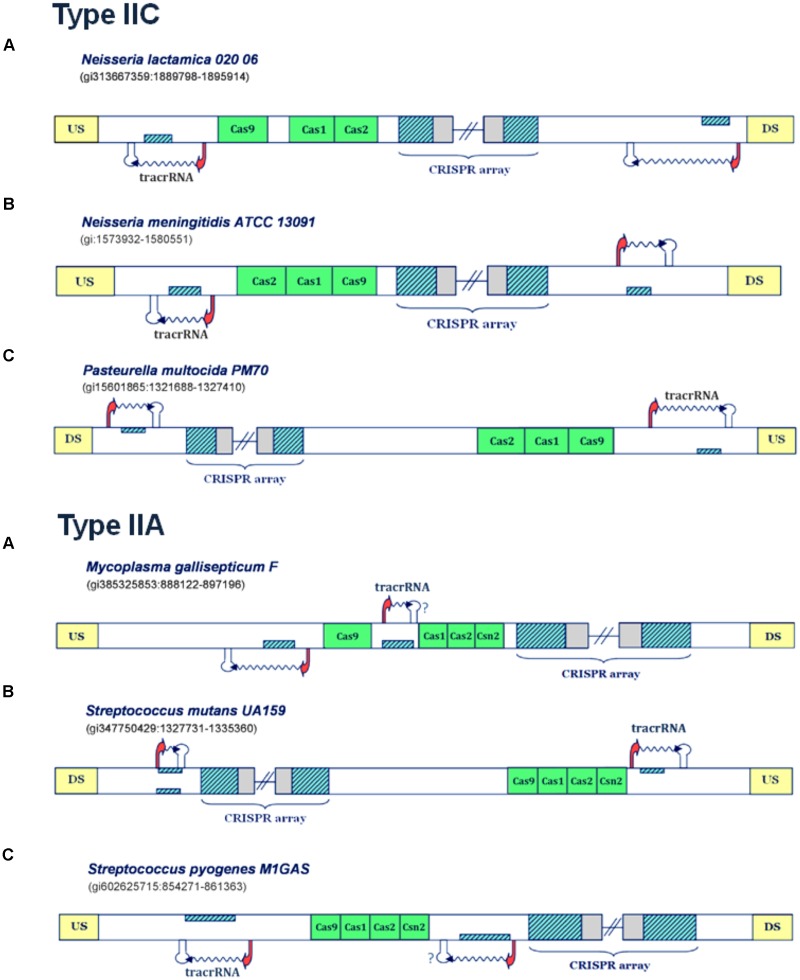
Predictions of scaRNA:tracrRNA pairs in Type IIC and IIA CRISPR/Cas systems. The organization of Type IIC systems found in *Neisseria lactamica 020 06*, *Neisseria meningitidis ATCC 13091* and *Pasteurella multocida PM70* is presented in the upper figure **(A–C)**, respectively; The organization of Type IIA systems found in *Mycoplasma gallisepticum F*, *Streptococcus mutans UA159* and *Streptococcus pyogenes M1GAS* is presented in lower figure **(A–C)**; The system components are marked analogously as in **Figure 1**.

**FIGURE 4:**
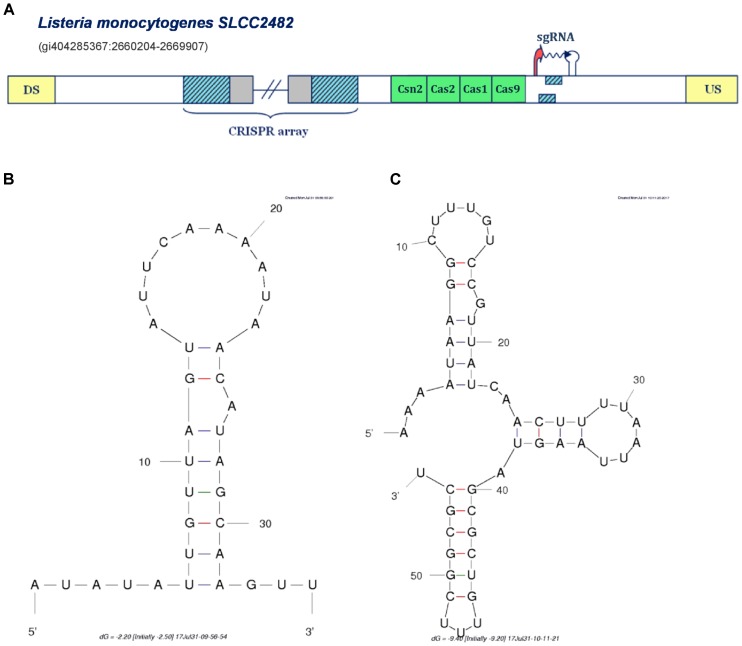
Prediction of sgRNA-like CRISPR/Cas-associated RNA in the IIA CRISPR/Cas system of *Listeria monocytogenes SLCC2428*. The organization of Type IIA system found in *Listeria monocytogenes SLCC2428* is presented in figure **(A)**; The system components are marked analogously as in **Figure 1**. **(B)** Secondary structure of the segment homologous to array repeat found in the Type IIA system of *L. monocytogenes SLCC2428*; **(C)** Secondary structure of the 3′ end of the predicted small RNA in the same CRISPR/Cas system.

In contrast to non-virulent strains analyzed in the previous section, the analysis of virulent strains in these subsystems, leads to clear predictions of scaRNA:tracrRNA pair, as shown in **Figures 3**, **4**, and further assessed below.

In **Figure 3A** (upper panel), the Type IIC system of *Neisseria lactamica 020 06* is presented, where the existence of two small RNA expression units can be inferred. Both of these units, one located upstream of the CRISPR array, and the other in the vicinity of *cas9* gene, are characterized by very strong promoter and terminator signals in “-” orientation. As the layout of transcription signals also suggests “-” oriented transcription for the CRISPR array, the unit predicted upstream from the array corresponds to scaRNA, since the segment homologous to repeat is found on the direct strand. Likewise, the segment homologous to repeat, located and expressed from the reverse strand (and upstream from *cas* genes), corresponds to tracrRNA. Similar expression pattern also occurs in Type IIC system of *Neisseria meningitidis ATCC 13091* (**Figure 3B**, upper panel), where the existence of two, analogously positioned small RNA expression units are inferred. Namely, both the expression of CRISPR array and putative tracrRNA (located in the vicinity of *cas* genes), occur from the reverse strand. The only difference with respect to *N. lactamica* is the expression of putative scaRNA, which is located upstream from the CRISPR array, and where the expression of inversely-oriented segment homologous to array repeat occurs from the direct strand. Finally, another example of a Type IIC system, with a prediction for scaRNA:tracrRNA pair, comes from bacterium *Pasteurella multocida PM70* (**Figure 3C**, upper panel). Two expression units, oriented in “+” direction, are observed upstream from the CRISPR array and *cas* genes, respectively. As the expression of CRISPR array probably occurs from the direct strand (see the predicted transcription start site signals in **Supplementary Table S1**), the *cas-*upstream expression unit, with the segment homologous to repeat, located on the reverse strand gives rise to tracrRNA, while the one located upstream from the array, with the segment homologous to repeat positioned on the direct strand, gives scaRNA.

Further on, the examples of the Type IIA systems with scaRNA:tracrRNA predictions are presented in the lower panel of **Figure 3**. For the Type IIA system of *Mycoplasma gallisepticum F* (**Figure 3A**, lower panel), the segments homologous to array repeat are found upstream from and between the *cas* genes, both located on the reverse strand. Due to close proximity of *cas* genes to the array, and also lack of downstream terminator signals, the expression of the *cas* genes and the array is likely coupled, thus occurring from the direct strand. Therefore, the segment homologous to repeat, located in between *cas* genes, which is preceded by its own promoter signal, corresponds to putative tracrRNA. A putative counterpart of tracrRNA (i.e., putative scaRNA) is found right upstream from *cas9* gene, where the expression of the inversely-oriented segment homologous to repeat occurs from the reverse strand.

Next, in **Figure 3B** (lower panel), we present the transcription scheme for the Type IIA system of *Streptococcus mutans UA159*, with two separate expression units located upstream from *cas* genes and in the vicinity of the array. As the promoter layout suggests the “-” oriented transcription of the array, the transcription unit, which is found in the vicinity of *cas* genes and harbors segment homologous to repeat, located and expressed from the direct strand, corresponds to putative tracrRNA. Analogously, the expression unit downstream from the array, with the “+” oriented expression of the inversely positioned segment homologous to repeat, corresponds to putative scaRNA.

Finally, *Streptococcus pyogenes M1GAS* (**Figure 3C**, lower panel) provides another example of a Type IIA system with two small RNA expression units. In this system, the layout of the predicted promoters suggests (coupled) “+” oriented transcription of *cas* genes and succeeding CRISPR array. Therefore, the “-” oriented transcription of the segment homologous to repeat, located on the reverse strand of the region that separates *cas* genes from the array, gives rise to putative scaRNA. Note that the terminator signal in this transcription orientation is missing, which, as stated previously, is most probably due to a false negative prediction. The tracrRNA that corresponds to this putative scaRNA is found upstream from *cas* genes, as a product of the inversely expressed segment homologous to array repeat, that is located on the direct strand.

### Possible Novel Mechanism of Small RNA Mediated CRISPR/Cas Activity

All the cases analyzed so far, correspond to the same mechanism of non-canonical Type II CRISPR/Cas functioning mediated through the activity of scaRNA:tracrRNA pairs, as experimentally established in *F. novicida U112*. In distinction, in **Figure 4A** we present a different scenario, predicted for the Type IIA system of *Listeria monocytogenes SLCC2428*, which may point to another paradigm of small RNA-mediated CRISPR/Cas activity. Namely, in the *cas-*upstream region of this system, we observe two overlapping segments homologous to array repeat, constrained by appropriately positioned transcription signals in the direct orientation.

The extent of mutual overlap indicates the possibility of complementary base-pairing between the 5′ and 3′ ends of the segments homologous to repeat, which we further corroborated by predicting RNA secondary structure corresponding to this sequence (see **Figure 4B**) (Zuker, 2003). Additionally, we also folded the flanking segment, that extends to the downstream terminator border, and obtained a structure with 3 succeeding stem-loops, remarkably alike to the known 3′-end structure of the tracrRNA molecule (see **Figure 4C**) (Nishimasu et al., 2014). Altogether, the spatial arrangement of this entire expression unit highly resembles the crRNA:tracrRNA/sgRNA duplex structure (Anders et al., 2014; Nishimasu et al., 2014).

In bioengineering applications, sgRNA makes an essential part of engineered Cas9-based constructs, which enables both the effector nuclease recruitment and target recognition within a single RNA molecule that mimics natural crRNA:tracrRNA duplex. Consequently, the transcription unit in *L. monocytogenes* Type IIA system may provide a functional equivalent of tracrRNA:scaRNA duplex within a single small RNA molecule. This new regulatory paradigm of jointly expressing both small RNAs, might be also well aligned with the virulence-associated activities, which usually constrain the system with narrow time frame to deliver proper physiological response, and consequently require a highly coordinated expression of the system components.

### When Available, RNA-Seq Data Are in Agreement With Predicted Small RNAs

We next check our predictions against available RNA-Seq data, which allows comparing our *ab initio* detection procedure with independent data (those in NCBI SRA database). In **Supplementary Table S2**, we summarize the existing supporting evidence, in terms of RNA-Seq data, for all predicted small RNA units. As can be seen, RNA-Seq data are scarce across diverse bacterial strains – e.g., except for *F. novicida U112* (the only strain with experimental confirmation for scaRNA) neither of the remaining Type IIB small RNAs could be compared against RNA-Seq data.

On the other hand, we successfully validated some of the predictions for scaRNA:tracrRNA pairs against RNA-Seq data, which provides an independent evidence for the paradigm on non-canonical Type II CRISPR/Cas functioning. Notably, as shown in **Supplementary Figure S2**, RNA-Seq data is in agreement with predictions in different strains (i.e., IIA systems of *S. pyogenes M1GAS* and *S. mutans UA159*), which otherwise lack the experimental support for scaRNA presence.

### Deeper Sequence Conservation Corroborates Predicted Small RNAs

Results of the conservation analysis for predicted CRISPR/Cas associated small RNAs are also summarized in **Supplementary Table S2**. As can be seen, most of the predicted small RNAs are conserved at the level of genus. However, in such cases, even by adjusting cut-off values, conservation of putative small RNA sequence does not stand out with respect to the flanking intergenic regions. This is illustrated in **Supplementary Figure S3**, in the case of experimentally established scaRNA and tracrRNA for *F. novicida U112*, which are conserved at the level of genus; note that regions related with scaRNA:tracrRNA pair function (indicated in the figure) also do not stand out as conserved. Consequently, conservation at the genus level is not a reliable predictor of CRISPR/Cas associated small RNAs, and these small RNAs may be highly variable. In support of this, it is interesting that, for putative CRISPR/Cas-associated small RNAs in Type IIB systems (for which direct experimental support is available), no conservation is found, or this conservation is only at the level of genus.

On the other hand, when conservation appears at larger phylogenetic distances, one can constrain the alignment to the region of predicted small RNA, as shown in **Supplementary Figure S4**. This corroborates accuracy of our *ab initio* procedure for small RNA detection, as the alignment boundaries match very well with predicted small RNA sequences (note that the information presented in **Supplementary Figures S3**, **S4** is also available in the **Supplementary Figure S1** in the form of a multiple alignment, followed by a corresponding conservation profile). Moreover, such deeper conservation (at class or phylum level), happens almost exclusively for tracrRNA (see the **Supplementary Table S2**). This is consistent with the fact that tracrRNAs are ubiquitous elements of Type II systems, where they have multiple roles (in addition to scaRNA mediated gene expression regulation). Moreover, this also corroborates that we accurately classify predicted small RNAs to scaRNA or tracrRNA categories, which is highly non-trivial, as this classification depends on predicted CRISPR array orientation – which in turn depends on prediction of the corresponding transcription signals. Finally, it is important to note that the predicted small RNA in the Type IIA system of *L. monocytogenes SLCC2482* (that resembles sgRNA) is well conserved (at class level), providing additional evidence for this prediction.

## Discussion

We performed the first computational investigation of the small CRISPR-associated RNAs in diverse Type II systems in the genomes of pathogenic bacteria. This study was motivated by the experimentally established connection between the Type II CRISPR/Cas components and bacterial virulence, with the underlying mechanisms still largely unknown (Louwen et al., 2013; Gunderson et al., 2015). Our reasoning was that the paradigm behind non-canonically acting Type IIB system of *F. novicida U112* (Sampson et al., 2013) – which is based on scaRNA:tracrRNA:Cas9 complex targeting mRNA – could be widespread platform for virulence-associated gene regulation by diverse Type II CRISPR/Cas systems, as the specificity for different targets is easily encoded in the sequence of guide RNA molecules.

In fact, it is becoming clearer that bacteria extensively exploit small non-coding RNAs to regulate the activity of endogenous genes, which are likely involved in processes central to cell physiology, including virulence in pathogenic strains (Toledo-Arana et al., 2007; Ahmed et al., 2016). Consequently, we analyzed 16 different Type II CRISPR/Cas systems of (mostly) pathogenic bacteria, where we showed the ubiquity of scaRNA:tracrRNA paradigm, which does not seem to apply to non-virulent strains; this, therefore, provides support to our initial hypothesis on putative virulence-related non-canonical CRISPR/Cas activities, mediated by the neighboring small RNAs.

In Type IIB systems, our predictions are consistent with the available experimental data, and we systematically identify scaRNAs throughout this subtype. Notably, in the Type IIB systems of *W. succinogenes DSM 1740*, *L. pneumophila 130b* and *F. novicida GA 99-3548*, our predictions for tracrRNA and, previously unrecognized, scaRNA units appeared in analogous positions as in *F. novicida U112* locus, though the corresponding genomic sequences of CRISPR loci are significantly different. This emerging uniform system architecture implies the ubiquity of the scaRNA species (and associated non-canonical system activities) throughout, at least, IIB subtype.

In distinction to subtype IIB, not all of the analyzed IIA/IIC systems are associated with unambiguous predictions for the scaRNA:tracrRNA pairs (see **Figure 2**). However, almost all loci without prediction for scaRNA reside within non-virulent strains supporting our proposal of associating scaRNA:tracrRNAs with virulence-related processes (Sampson et al., 2013). Namely, the pathogenicity of the strain *H. parainfluenzae T3T1*, where the only expression unit corresponds to the *cas*-upstream positioned tracrRNA, is rarely reported, even though it is closely related to the well-known pathogen of the respiratory tract, *H. influenzae*. Likewise, *L. innocua CLIP11262*, with analogously positioned tracrRNA, represents a non-pathogenic strain, although it is closely related to pathogenic *L. monocytogenes* species. Moreover, *L. salivarius UCC118*, whose CRISPR/Cas locus also harbors prediction for only tracrRNA, is a useful probiotic bacterium that interferes with pathogenic strains within gastrointestinal tract.

The only strain that does not fit into this paradigm is *C. jejuni 81116*, where in addition to virulence, the involvement of Type II CRISPR/Cas components during infection establishment was also evidenced (Louwen et al., 2013). The fact that we predict only tracrRNA in the IIC locus of this strain implies that a distinct mechanism or system architecture might be employed for delivering virulence-related non-canonical activities in this case – e.g., scaRNAs encoded outside the CRISPR/Cas locus. In fact, the involvement of external components was already well-established for the canonical Type II immune functions, as RNAse III provides indispensable contribution to crRNA/tracrRNA maturation during system expression (Deltcheva et al., 2011).

Apart from *C. jejuni 81116*, we were consistently able to infer scaRNA:tracrRNA pairs throughout CRISPR/Cas systems of the remaining pathogenic strains (see **Figure 3**). Notably, one of these scaRNA-harboring systems belongs to pathogenic *N. meningitidis* species (**Figure 3B**, upper panel), where the connection between CRISPR/Cas components and virulence was experimentally established; note that such connection was already discussed for the strain *L. pneumophila 130b*, whose Type IIB system also accommodates putative scaRNA unit. These predictions strongly suggest virulence-related activities for small CRISPR-associated RNA units; however, this relation cannot always be straightforwardly asserted, as we also predict scaRNA in some non-virulent strains (precisely, *N. lactamica 020 06* and *W. succinogenes DSM 1740*), implying that non-canonical CRISPR/Cas activities in some cases might affect cellular processes beyond those related to virulence.

The seemingly common non-canonical functioning of Type II CRISPR/Cas systems might be favored by the coexistence of multiple CRISPR/Cas systems per bacterial genome, which we encounter throughout the genomes of e.g., *S. pyogenes M1GAS*, *F. novicida U112*, *P. multocida PM70*, all harboring putative scaRNA units. As previously proposed (Ratner et al., 2015), one of CRISPR/Cas loci may take over defense-related activities, so that the remaining systems may influence other aspects of cellular physiology by resorting to non-canonical functions. Related to this, potentially harmful side-effects (on bacterial fitness) that could arise as a consequence of the system immune function, in particular due to reduction of HGT, could be compensated by non-canonical functions, e.g., through reinforcing virulence.

We noted previously that the predicted small RNA expression units, which are at the core of non-canonical CRISPR/Cas functioning, are sometimes deprived of succeeding terminator signals. Note also that our inferred transcription scenarios are sometimes deprived of promoter signals too (see e.g., the schemes in **Figure 2** for *H. parainfluenzae T3T1* and *L. salivarius UCC118*), which is probably due to existence of weak promoter elements, which fall below the search threshold. These cryptic elements might be under external regulatory control (e.g., transcription factor control), to enable activation only under narrow-ranged conditions; likewise, the expression of system components might be also coupled with promoters of alternative σ factors [e.g., ECF σ factors signalizing envelope stress (Helmann, 2002)], which are known to be induced under highly stringent conditions, whereby producing rapid responses to the activating stimuli (Staron et al., 2009). Such complications with detecting transcription signals also underline the complex nature of predicting scaRNA:tracrRNA pairs (also noted in Introduction), which we, in this first instance, resolved in part through manual curation.

Conservation analysis showed that significant alignments are usually restricted to closely related species, in line with common notion of small RNAs not being well conserved in genome sequences, and with involvement of CRISPR-associated small RNAs in virulence (typically related with highly variable genome regions). Moreover, predictions that are more deeply conserved are consistently annotated as tracrRNAs, which is consistent with dual role (in both canonical and non-canonical functioning) that they have in the system. In addition to tracrRNAs, the predicted sgRNA-like CRISPR/Cas associated small RNA, was also well conserved (at the level of class), whose compact design might present a novel paradigm for exerting non-canonical CRISPR/Cas functions. Lower level of conservation associated with scaRNAs, may indicate that even closely related strains can explore different avenues to impact virulence through non-canonical CRISPR/Cas activities, which is further supported by the observation that the closely related species/strains commonly display varying levels of virulence (Sampson and Weiss, 2013). In other words, non-canonical functions mediated by CRISPR/Cas may have been hijacked more recently, as already discussed above in the context of Cas1-2 absence in *F. novicida GA99-3548*.

One such hijacking, leading to scaRNAs, might be provided by remnants of CRISPR array acquiring a new function, where a direct array repeat may be partially preserved within a newly formed transcription unit. In particular, it was proposed that this is what happened in the experimentally established case of *F. novicida* U112 (Chylinski et al., 2014). On the other hand, to prevent falsely reporting (recently) degenerated remnants of CRISPR array as scaRNAs, for each of our predictions we checked that there are no additional segments homologous to the direct repeats, between the predicted small RNAs and CRISPR array. Also, to prevent reporting small CRISPR arrays as CRISPR/Cas associated small RNAs, we checked for the direct repeat homology regions closely spaced with respect to each other.

Also, regarding conservation, it is interesting that we didn’t observe virtually any conservation of tracrRNA region established to recognize its mRNA target in *F. novicida U112*. This indicates that targets of scaRNA:tracrRNA-Cas9 may also be highly variable, complicating their computational identification. Namely, our attempt to computationally recover experimentally identified target failed, as we obtained too large number of hits due to too short region of homology with mRNA target. Likely low conservation of the targets, would prevent using it to further filter these hits. Therefore, computational recognition of scaRNA:tracrRNA targets remains a significant (and likely complicated) problem to be addressed in the future. Further outlook of this work is presented in the next section.

## Summary and Outlook

Few independent lines of evidence, including recovering small RNAs in a subtype with existing experimental evidence, conservation analysis, and mining RNA-Seq data, show that *ab initio* predictions directly from genome sequence – based primarily on identifying relevant transcription signals – is an optimal approach for large-scale predictions of CRISPR/Cas-associated small RNAs. With regard to alternative approaches, we obtain that those based primarily on mining RNA-Seq data, or conservation analysis, would not be suitable, due to current scarcity of RNA-Seq data or generally low conservation of CRISPR/Cas-associated small RNAs. However, in few cases where RNA-Seq data were available, RNA-Seq reads showed significant alignment with predicted scaRNA:tracrRNA pairs. Overall, our analysis indicates that scaRNA:tracrRNA paradigm could be exploited in diverse Type II systems of pathogenic bacteria, thus acting as a common framework for non-canonical system activities that influence virulence-related processes. Our predictions also suggest a possibility for a somewhat different mechanism compared to the experimentally established scaRNA:tracrRNA duplex, exhibited through a single sgRNA-like unit, which we also found to be well conserved. Moreover, our method enables functional assignment of the predicted expression units (i.e., distinguishing scaRNAs from tracrRNAs), which can aid prediction of putative endogenous targets of these small RNAs, and consequently promote further understanding of this framework. However, as discussed above, predicting scaRNA:tracrRNA targets is a non-trivial problem to be addressed in the future.

Another important outlook for future research is developing an automated predictor for CRISPR/Cas-associated small RNAs, which would allow their large-scale analysis (e.g., across all sequenced strains with Type II systems). This is in distinction to the present study, where a careful manual curation of a relatively smaller number of CRISPR/Cas loci, chosen to cover all three Type II subtypes and to include literature examples where CRISPR/Cas was related to virulence, was performed. Regarding this, the present study can be viewed as a starting point, needed for both future experiments that address the important issue of non-canonical CRISPR/Cas functions, and also for providing a necessary training set for developing the automated predictor. Such predictor would also allow an empirical estimate of significance of small RNA predictions, without necessity of calibrating them on experimentally supported subtypes, or cross-checking them with RNA-Seq data and homology searches, as done in this study. That can be done through a brute-force approach, e.g., by estimating number of hits in random sequences, since prediction of CRISPR/Cas-associated small RNAs involves an interrelation between a number of individual elements, which otherwise largely complicates significance estimate. The large scale analysis by an automated search procedure would also allow to more firmly establish a link between scaRNA:tracrRNA paradigm and bacterial virulence, which is now implied by both our study and (mainly indirect) experimental evidence.

Finally, while this study concentrated on Type II systems (due to its experimentally established link with bacterial virulence), an evident future extension of this study would be to also explore this link in other CRISPR-Cas types, in particular in major Type I and Type III systems. This is, however, a highly non-trivial goal, as the mechanism for exhibiting non-canonical functions may be different than the one in Type II systems, mostly because Type I and Type III systems do not involve tracrRNAs. For example, a recent study showed a regulation of virulence processes by Type I-F CRISPR/Cas systems, where this mechanism is exhibited through crRNA targeting a host mRNA (canonically, crRNA targets foreign DNA; Li et al., 2016). This experimental evidence in Type I system, even-more underlines importance of this work, as it points out to a widespread relationship between CRISPR/Cas and bacterial virulence, transcending Type II systems. We therefore think that this study presents an important first step toward a wider and more systematic understanding of a relation between CRISPR/Cas and bacterial pathogenicity, which in turn might be of a groundbreaking medical and biological importance.

## Author Contributions

All authors have given approval to the final version of the manuscript. MarD conceived the work, with the help of MagD, EZ and JG. JG implemented the method with the help of MagD. JG, W-HC, and TS performed the analysis. All the authors interpreted the results. JG wrote the paper, with the help of MarD, MagD, W-HC, and EZ.

## Conflict of Interest Statement

The authors declare that the research was conducted in the absence of any commercial or financial relationships that could be construed as a potential conflict of interest.
