# Supplementary material for: In silico Analysis Suggests Common Appearance of scaRNAs in Type II Systems and Their Association With Bacterial Virulence
Source: Front Genet. 2018 Oct 17;9:474. doi: 10.3389/fgene.2018.00474 (PMC6199352; doi:10.3389/fgene.2018.00474)
Supplement: Supplementary file 1 [file Table_1.DOCX]

Supplementary Material

***In-silico* analysis suggests common appearance of scaRNAs in Type II systems and their association with bacterial virulence**

**Jelena Guzina, Weihua Chen, Tamara Stankovic, Magdalena Djordjevic, Evgeny Zdobnov, Marko Djordjevic***

*** Correspondence:** Marko Djordjevic: dmarko@bio.bg.ac.rs

# Supplementary Table S1. The exact scores, coordinates and CRISPR/Cas locus positions for all of the obtained search hits.

| **Bacterial strain** | **Intergenic region** | **Promoter scores and coordinates** | **Terminator scores and coordinates** | **Direct repeat match coordinates** |
| --- | --- | --- | --- | --- |
| Campyilobacter jejuni 81116 | ***cas*-array direct** | -2,1795; 69  -6,7925; 64 | -11,50; 108-143  -9,60; 32-79 |  |
|  | ***cas*-array reverse** | -5,7723; 45  -6,2702; 90  -6,6605; 93  -6,9891; 105 | -12,60; 11-60 | 48-83 |
|  | **array-DS direct** | -6,7047; 46 | -8,80; 89-115 |  |
|  | **array-DS reverse** |  | -6,40; 11-50 |  |
| Francisella novicida GA99-3548 | ***cas9* upstream direct** | -5,8180; 451  -6,2090; 483  -6,7449; 341  -6,7797; 266  -6,9183; 431  -7,0012; 322  -7,0633; 289 | -8,40; 164-204  -7,50; 149-197 | 64-102 |
|  | ***cas9* upstream reverse** | -2,3434; 440  -5,6863; 255  -6,1084; 148  -6,8272; 540  -6,9617; 457 | -8,20; 515-560 | 107-147 |
|  | ***cas9-cas4* direct** | -7,0354; 85 |  | 62-98 |
|  | ***cas9-cas4* reverse** | -4,8699; 120  -5,9033; 113  -6,4637; 133  -6,9461; 92  -6,9562; 71 |  | 79-117 |
|  | ***cas4*-array direct** | -5,6840; 290  -6,3355; 271  -6,3949; 243 | -10,40; 127-157  -8,40; 565-609 | 110-142 |
|  | ***cas4*-array reverse** | -3,6905; 50  -4,4552; 116  -6,9795; 251  -6,9936; 347 | -10,70; 203-233  -7,90; 261-291  -7,30; 12-51 | 108-148 |
|  | **array-DS direct** | -5,3759; 67  -5,8164; 463  -6,3956; 109 | -8,80; 584-630 | 170-208 |
|  | **array-DS reverse** | -4,5861; 154  -4,6422; 286  -4,6760; 655  -5,4290; 701  -5,6910; 447  -5,7049; 606  -5,7509; 459  -5,7556; 663  -5,8517; 242  -6.0068; 339  -6,0301; 442  -6,3030; 469  -6,3916; 298  -6,4385; 651  -6,5834; 402  -6,6945; 48  -6,8501; 633  -6,8637; 285  -6,9462; 171 | -7,40; 340-387 | 239-274 |
| Haemophilus parainfluenzae T3T1 | ***cas* upstream direct** | -6,5636; 113  -6,6690; 294 | -15,10; 45-78 |  |
|  | ***cas* upstream reverse** | -2,9986; 96  -6,7457; 80  -6,9613; 69 | -20,00; 160-201  -13,30; 252-281 | 105-139 |
|  | ***cas-*array direct** | -7,0895; 35 | -13,70; 21-49 |  |
|  | ***cas-*array reverse** |  | -20,50; 11-43 |  |
|  | **array-DS direct** |  | -21,50; 18-57 |  |
|  | **array-DS reverse** |  | -23,80; 12-49 |  |
| Listeria innocua CLIP11262 | ***cas* upstream direct** | -3,1427; 58  -6,4536; 67  -6,9749; 48 | -14,70; 404-423  -12,40; 342-356  -9,30; 132-148 | 61-96 |
|  | ***cas* upstream reverse** | -4,9845; 164  -5,3437; 140  -5,3437; 237  -6,2485; 380  -6,4535; 352 | -19,20; 13-38 | 359-393 |
|  | ***cas-*array direct** | -6,0624; 99 |  |  |
|  | ***cas-*array reverse** | -5,8280; 52 |  |  |
| Mycoplasma gallisepticum F | ***cas9* upstream direct** | -4,5915; 202  -4,7300; 192  -5,6398; 417  -6,2319; 144  -6,5627; 39  -7,0156; 50 | -7,60; 368-415 |  |
|  | ***cas9* upstream reverse** | -6,2981; 68  -6,7034; 99 | -9,40; 202-250 | 65-101 |
|  | ***cas9-cas* direct** | -3,0081; 57  -6,6613; 40 |  |  |
|  | ***cas9-cas* reverse** |  |  | 13-45 |
|  | **array-DS reverse** | -6,1041; 55 |  |  |
| Neisseria lactamica 020 06 | ***cas9* upstream direct** | -5,7895; 246 |  | 151-187 |
|  | ***cas9* upstream reverse** | -2,6405; 104  -5,6856; 52  -6,5514; 149 | -29,40; 228-269  -25,80; 173-214 | 112-146 |
|  | ***cas-*array direct** |  | -22,80; 6-40 |  |
|  | **array-DS direct** | -6,3236; 302 | -15,50; 190-226  -15,20; 248-273  -14,40; 547-593  -14,30; 338-377  -11,70; 372-404 | 450-484 |
|  | **array-DS reverse** | -3,7150; 59  -3,7187; 390  -5,4138; 548  -5,8092; 377  -6,8174; 495 | -16,50; 487-521  -12,00; 411-451  -10,30; 326-345 | 236-273 |
| Neisseria meningitidis ATCC 13091 | ***cas* upstream direct** | -5.7895; 245  -6.8787; 183 |  | 150-184 |
|  | ***cas* upstream reverse** | -2.6405; 103  -3.9721; 51 | -25,80; 172-213  -24,50; 227-267 | 111-145 |
|  | ***cas-*array reverse** |  | -22,90; 6-40 |  |
|  | **array-DS direct** | -2.7815; 200  5.7497; 34 | -20,10; 299-344  -14,50; 401-432  -14,00; 91-139 | 34-70 |
|  | **array-DS**  **reverse** |  |  |  |
|  |  | -6.7624; 367 | -17,70; 306-344 | 204-236 |
| Pasteurella multocida PM70 | ***cas* upstream direct** | -4,9437; 98  -5,7500; 49  -6,9945; 67 | -22,60; 166-208 | 104-141 |
|  | ***cas* upstream reverse** | -5,7387; 58  -6,6913; 184 |  | 84-119 |
|  | ***cas-*array direct** | -6,2141; 187  -6,4808; 93  -6,5916; 182 | -22,70; 8-38  -6,80; 167-205 | 144-185 |
|  | ***cas-*array reverse** | -5,5083; 154 |  | 183-216 |
|  | **array-DS**  **direct** | -4,9656; 67  -5,9882; 388  -6,4764; 415  -6,6330; 243  -6,7816; 436 | -6,00; 297-342 | 190-226 |
|  | **array-DS**  **reverse** | -4,8419; 35  -6,4464; 224 | -14,70; 123-167  -10,70; 339-384  -8,00; 290-339  -7,10; 231-256 | 238-277 |
| Streptococcus mutans UA159 | ***cas* upstream direct** | -2,3256; 56  -5,5282; 350  -6,1468; 363  -6,6875; 229  -6.8031; 194  -6,9296; 274 | -8,00; 143-157 | 60-94 |
|  | ***cas* upstream reverse** | -2,3513; 195  -5,1115; 151  -5,1854; 74  -5,7462; 124  -5,7630; 173  -6,2961; 338 | -14,50; 15-56 | 215-248 |
|  | ***cas-*array direct** | -4,4504; 257  -5,6286; 163  5,7694; 555  -5,8504; 110  -6,3045; 203  -6,3931; 536  -6,5916; 635  -6,6106; 108  -6,6872; 526  -6,9736; 479 | -16,90; 115-148  -7,70; 205-252  -7,30; 296-335  -6,90; 699-747 | 212-247 |
|  | ***cas-*array reverse** | -2,4365; 736  -2,6854; 81  -4,8620; 49  -4,9908; 70  -5,1439; 282  -6,2269; 209  -6,2831; 696  -6,4945; 317  -7,0036; 614 | -16,80; 691-722  -11,1; 13-39  -10,30; 476-518 | 485-517 |
|  | **array-DS**  **direct** | -5,2852; 82  -5,5511; 109 | -17,80; 19-55  -7,80; 96-136 | 84-118 |
|  | **array-DS**  **reverse** |  | -14,90; 98-126 | 31-68 |
| Streptococcus pyogenes M1GAS | ***cas* upstream direct** | -5,3760; 163  -5,8472; 397  -6,2239; 322  -6,6193; 91  -6,7463; 439  -6,9482; 204  -7,0166; 278 | -14,30; 37-65  -6,10; 139-171 | 154-188 |
|  | ***cas* upstream reverse** | -3,1217; 288  -3,9859; 207  -6,0203; 176  -6,5399; 96  -6,6095; 330  -7,1028; 277 | -10,80; 415-443  -10; 359-375 | 381-415 |
|  | ***cas-*array reverse** | -6,3399; 45 |  | 46-81 |
| Listeria monocytogenes SLCC 2428 | ***cas* upstream direct** | -3.1427; 58  -5.3386; 307  -5.5077; 437  -5.8209; 48  -6.1476; 296  -6.1663; 285  -6.2831; 182  -6.4536; 67  -6.6114; 211  -6.8759; 326  -6.9393; 349  -6.9759; 216  -7.0878; 191 | -13,9; 369-406  -9,8; 443-485  -9,3; 132-148 | 61-96 |
|  | ***cas* upstream reverse** | -5.3384; 238  -5.4494; 156  -6.2485; 451  -6.4535; 423  -6.5511; 39  -6.5661; 86  -6.7688; 233  -6.9059; 180  -6.9138; 353  -7.0451; 214 | -14,8; 104-141  -7,3; 161-196 | 430-464 |
|  | ***cas-*array direct** | -4.5426; 100 | -6,4; 50-86 |  |
|  | ***cas-*array reverse** | -5.1992; 46 |  |  |
|  | **array-DS**  **direct** | -5.1290; 111  -5.8709; 213  -6.7030; 90  -6.8343; 105  -6.9787; 218 | -9,2; 116-158  -7; 4-53 |  |
|  | **array-DS**  **reverse** | -4.7970; 421  -6.4895; 502  -6.6925; 490  -6.7125; 42 | -8; 109-130 | 489-525 |
| Francisella novicida U112 | ***cas-*array direct** | -5.2892; 272  -5.6840; 291  -6.3949; 244  -6.4051; 114 | -10,4; 127-157  -8,4; 164-204 |  |
|  | ***cas-*array reverse** | -3.6905; 51 | -10,7; 203-233  7,9; 261-291  7,3; 12-51  6,4; 331-352 | 108-145 |
|  | **array-DS**  **direct** | -5.3759; 68  -6.3956; 110 |  | 170-207 |
|  | **array-DS**  **reverse** | -4.5861; 155  -4.6422; 287  -4.676; 654  -5.429; 702  -5.691; 448 | -7,4; 340-387  -7; 599-633 |  |
| Legionella pneumophila 130b | ***cas-*array direct** | -3.7406; 214  -5.7666; 197  -6.7387; 92  -6.9069; 127 | -8; 68-110 |  |
|  | ***cas-*array reverse** | -7.0936; 85 | 15,8; 174-213 | 119-157 |
|  | **array-DS**  **direct** |  | -10,6; 76-124 | 39-76 |
|  | **array-DS**  **reverse** | -4.5357; 277  -4.8842; 104  -5.4234; 267  -5.727; 137  -6.4137; 117 | 9,4; 303-351  -6.1; 378-409 |  |
| Wolinella succinogenes DSM 1740 | ***cas-*array direct** | -6.754; 140  -6.79; 43 | -8; 42-72  -14,2; 109-156 |  |
|  | ***cas-*array reverse** | -6.241; 88 | -17; 56-103  -12,7; 180-206 | 122-159 |
|  | **array-DS**  **direct** |  |  | 38-75 |
|  | **array-DS**  **reverse** | -5.5753; 170  -7.029; 90  -7.0317; 131 | -8,1; 115-156 |  |
| Lactobacillus salivarius UCC118 | ***cas9* upstream direct** | -4,4953; 140  -4,7860; 95 | -13,60; 30-58 |  |
|  | ***cas9* upstream reverse** |  | -6,80; 113-135 |  |
|  | ***cas9-cas* direct** | -3,6621; 271  -5,1333; 393  -5,7377; 64  -6,7480; 311  -6,9519; 127  -7,0340; 274 | -13,40; 4-36  -11,90; 595-614 | 676-713 |
|  | ***cas9-cas* reverse** | -2,7138; 42  -4,3723; 375  -4,8508; 603  -5,0195; 359  -5,0502; 653  -5,6529; 605  -5,8740; 723  -6,0539; 647  -6,4922; 756  -6,6678; 674  -6,8313; 434  -6,9615; 534 | -17,00; 144-173  -7,10; 71-120  -6,60; 236-273 | 23-59 |
|  | **array-DS**  **reverse** | -6,5604; 59  -6,7091; 91 |  |  |

**2 Supplementary Table S2.** **Validation of small CRISPR-associated RNA *ab initio* predictions**

| **Bacterial strain** | **Predicted small RNA units**  - genomic coordinates - | **RNA-Seq** | **Conservation analysis**  - taxonomy rank - |
| --- | --- | --- | --- |
| ***Type IIA*** | | | |
| *S. pyogenes M1GAS* | **tracrRNA**: c(854308:854463)  **scaRNA**: c(860716:860774) | **yes**  **yes** | **yes; genus**  **yes; genus** |
| *L. innocua CLIP11262* | **tracrRNA**: 2774769:2774859 | **yes** | **yes; class** |
| *L. monocytogenes SLCC2428* | **sgRNA**: 2669455:2669545 | **no** | **yes; class** |
| *L. salivarius UCC118* | **tracrRNA**: c(118779:118910) | **yes** | **yes; genus** |
| *S. mutans UA159* | **tracrRNA**: 1335035:1335136  **scaRNA**: 1327813:1327867 | **yes**  **yes** | **yes; genus**  **yes; genus** |
| *M. gallisepticum F* | **tracrRNA**: 892451:892478  **scaRNA**: c(888352:888534) | **no**  **no** | **no**  **no** |
| ***Type IIB*** | | | |
| *F. novicida U112* | **tracrRNA**: c(817027:817209)  **scaRNA**: c(818307:818653) | **yes**  **yes** | **yes; genus**  **yes; genus** |
| *F. novicida GA99-3548* | **tracrRNA**: c(397990:398057)  **scaRNA**: c(398938:399198) | **no**  **no** | **yes; genus**  **yes; genus** |
| *W. succinogenes DSM 1740* | **tracrRNA**: c(1531720:1531838)  **scaRNA**: c(1533495:1533556) | **no**  **no** | **no**  **no** |
| *L. pneumophila 130b* | **tracrRNA**: c(210316:210444)  **scaRNA**: c(214748:214898) | **no**  **no** | **no**  **no** |
| ***Type IIC*** | | | |
| *H. parainfluenzae T3T1* | **tracrRNA**: c(1913134:1913239) | **no** | **yes; phylum** |
| *C. jejuni 81116* | **tracrRNA**: 1441284:1441358 | **yes** | **yes; genus** |
| *N. meningitidis ATCC 13091* | **tracrRNA**: c(1573997:1574107)  **scaRNA**: 1580312:1580456 | **no**  **no** | **yes; phylum**  **yes; genus** |
| *N. lactamica 020 06* | **tracrRNA**: c(1889864:1890033)  **scaRNA**: c(1895569:1895855) | **yes**  **only a few hits** | **yes; phylum**  **yes; genus** |
| *P. multocida PM70* | **tracrRNA**: 1327284:1327394  **scaRNA**: 1321755:1322030 | **no**  **no** | **yes; phylum**  **no** |
