# Supplementary material for: In silico Analysis Suggests Common Appearance of scaRNAs in Type II Systems and Their Association With Bacterial Virulence
Source: Front Genet. 2018 Oct 17;9:474. doi: 10.3389/fgene.2018.00474 (PMC6199352; doi:10.3389/fgene.2018.00474)
Supplement: Supplementary file 2 [file Data_Sheet_1.pdf]

Francisella novicida U112 (tracrRNA)

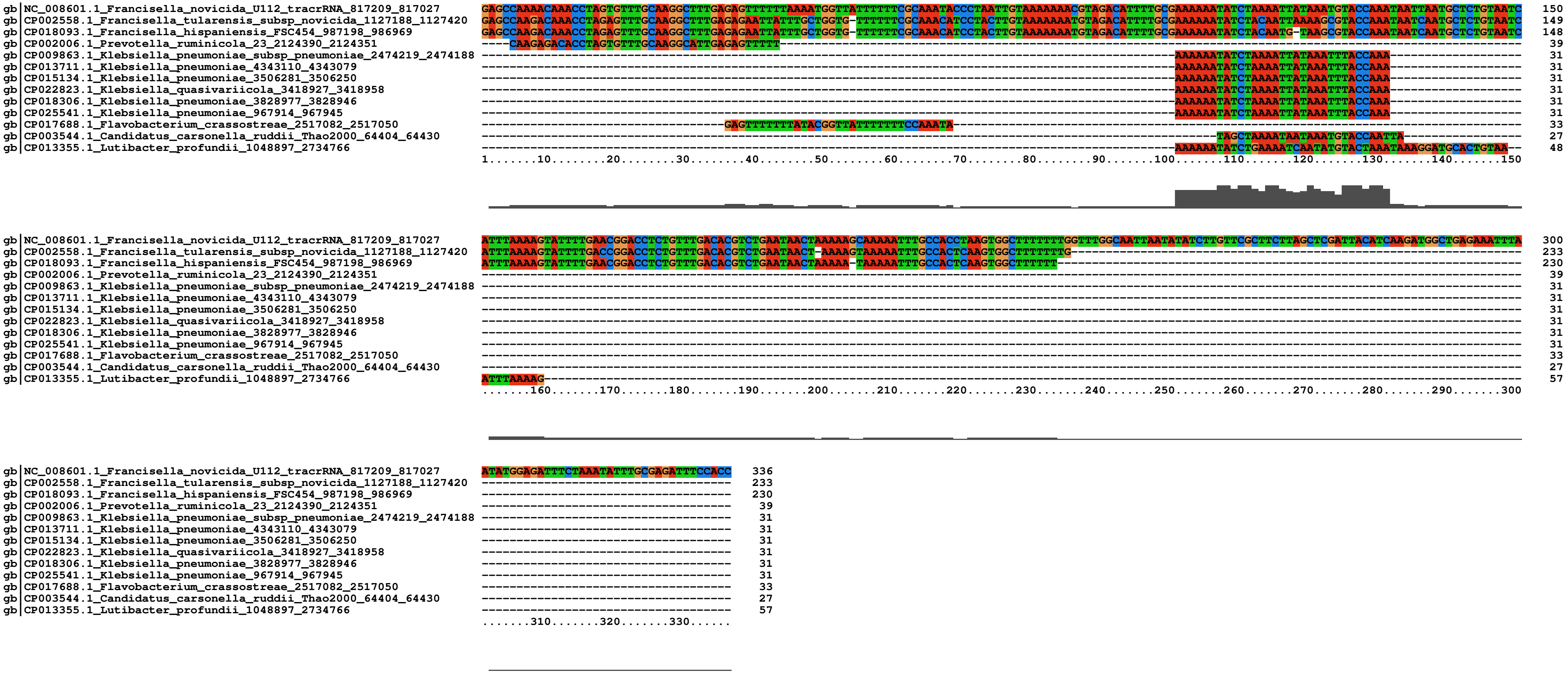

Supplementary Figure S1. MSA and conservation profiles for small RNAs and their flanking regions shown in Figs. S3 and S4. Multiple sequence alignments (MSA) and the corresponding conservation profiles (generated by BLAST and ClustalX), corresponding to more deeply conserved small RNAs and their flanking regions (shown in Figs. S3 and S4), are presented. For all sequences, their accession numbers, strain of origin, and genomic coordinates of the aligned regions are indicated. Strains of origin for small RNA with flanking regions (corresponding to the names indicated in Figs. S3 and S4), with respect to whom all the hits are aligned, are shown above each MSA. It can be seen that for predicted small RNAs with conservation level at larger phylogenetic distances (all small RNAs except F. novicida U112 tracrRNA and scaRNA), the alignment (conserved region) is restricted to small RNA, while the flanking segments are not conserved.

Francisella Novicida U112 (scaRNA)

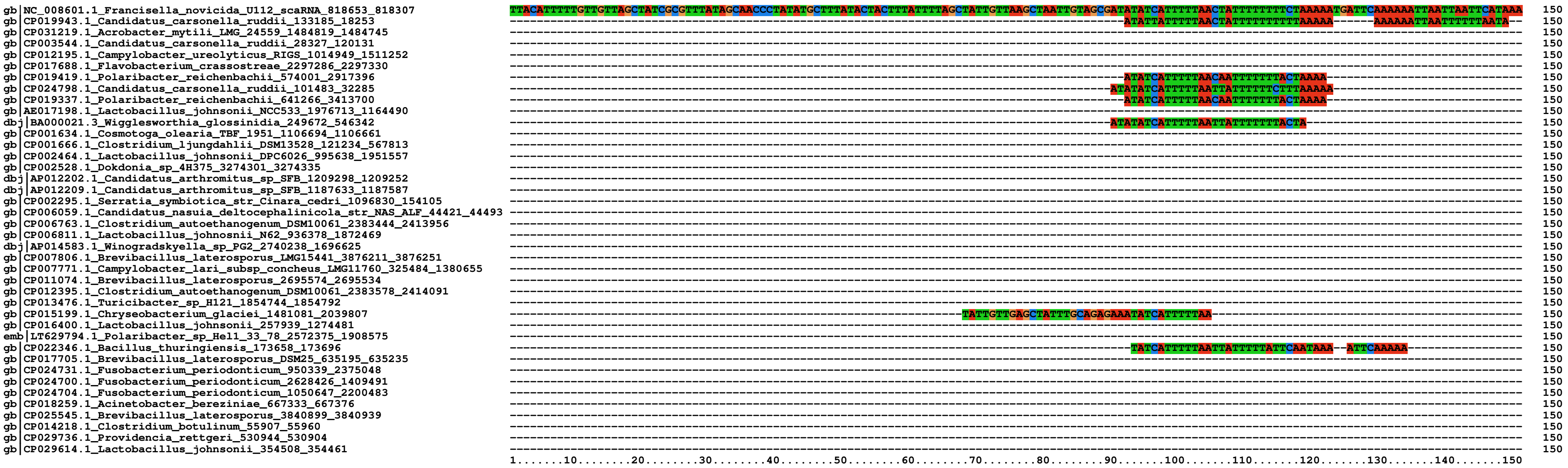

Francisella Novicida U112 (scaRNA)

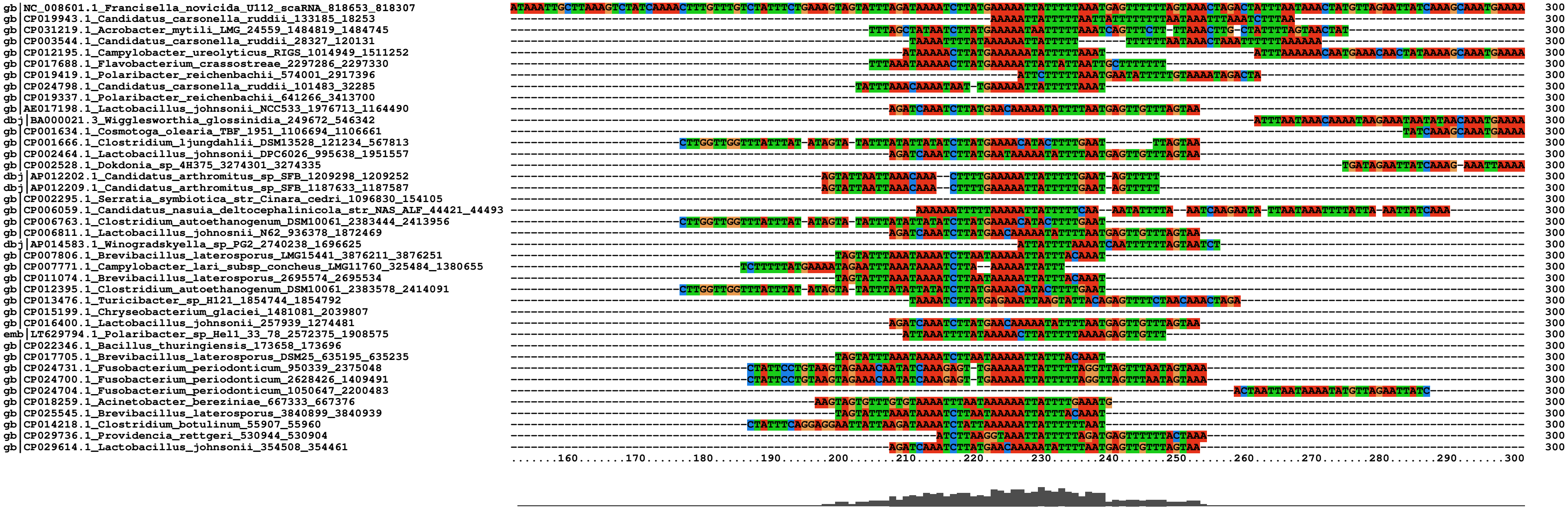

Francisella Novicida U112 (scaRNA)

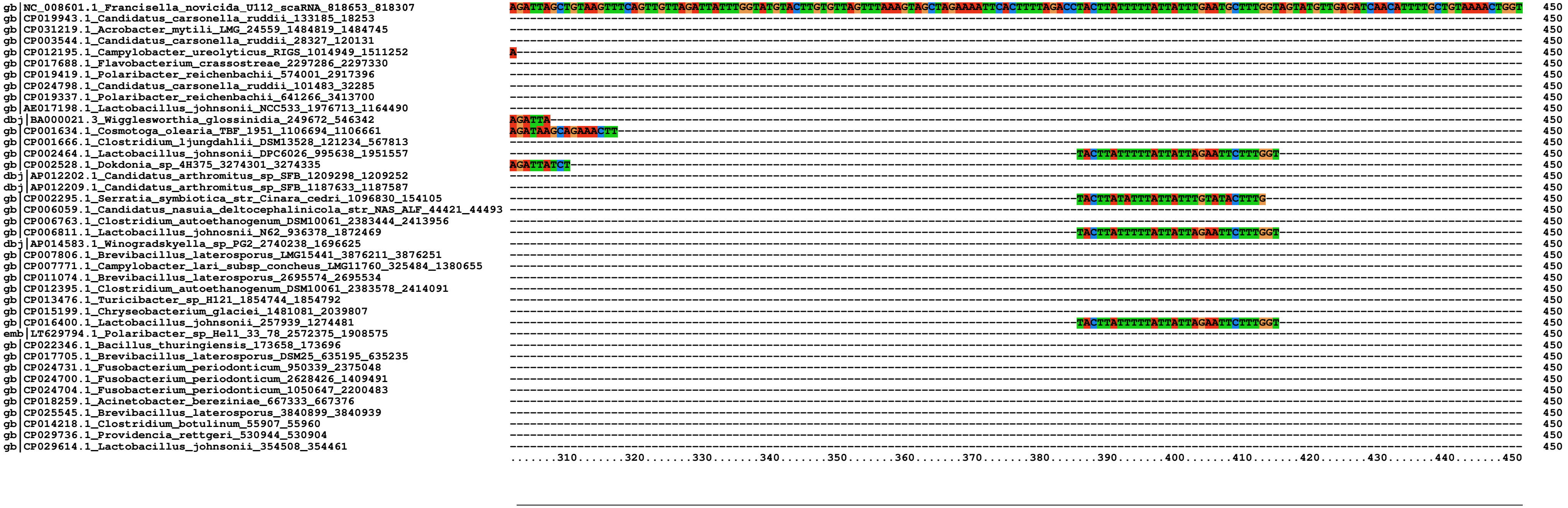

Francisella Novicida U112 (scaRNA)

```
gb|NC_008601.1_Francisella_novicida_U112_scaRNA_818653_818307                TTTCGTGATTATATTATTTGATACAATGTGTCCAACTAGAAACTTTTAGGAATAATTTACAAAATTAATTAAG   523
gb|CP019943.1_Candidatus_carsonella_ruddii_133185_18253                      -------------------------------------------------------------------------   523
gb|CP031219.1_Acrobacter_mytili_LMG_24559_1484819_1484745                    -------------------------------------------------------------------------   523
gb|CP003544.1_Candidatus_carsonella_ruddii_28327_120131                      -------------------------------------------------------------------------   523
gb|CP012195.1_Campylobacter_ureolyticus_RIGS_1014949_1511252                 -------------------------------------------------------------------------   523
gb|CP017688.1_Flavobacterium_crassostreae_2297286_2297330                    -------------------------------------------------------------------------   523
gb|CP019419.1_Polaribacter_reichenbachii_574001_2917396                      -------------------------------------------------------------------------   523
gb|CP024798.1_Candidatus_carsonella_ruddii_101483_32285                      -------------------------------------------------------------------------   523
gb|CP019337.1_Polaribacter_reichenbachii_641266_3413700                      -------------------------------------------------------------------------   523
gb|AE017198.1_Lactobacillus_johnsonii_NCC533_1976713_1164490                 -------------------------------------------------------------------------   523
dbj|BA000021.3_Wigglesworthia_glossinidia_249672_546342                      -------------------------------------------------------------------------   523
gb|CP001634.1_Cosmotoga_olearia_TBF_1951_1106694_1106661                     -------------------------------------------------------------------------   523
gb|CP001666.1_Clostridium_ljungdahlii_DSM13528_121234_567813                 -------------------------------------------------------------------------   523
gb|CP002464.1_Lactobacillus_johnsonii_DPC6026_995638_1951557                 -------------------------------------------------------------------------   523
gb|CP002528.1_Dokdonia_sp_4H375_3274301_3274335                              -------------------------------------------------------------------------   523
dbj|AP012202.1_Candidatus_arthromitus_sp_SFB_1209298_1209252                 -------------------------------------------------------------------------   523
dbj|AP012209.1_Candidatus_arthromitus_sp_SFB_1187633_1187587                 -------------------------------------------------------------------------   523
gb|CP002295.1_Serratia_symbiotica_str_Cinara_cedri_1096830_154105            -------------------------------------------------------------------------   523
gb|CP006059.1_Candidatus_nasuia_deltocephalinicola_str_NAS_ALF_44421_44493   -------------------------------------------------------------------------   523
gb|CP006763.1_Clostridium_autoethanogenum_DSM10061_2383444_2413956           -------------------------------------------------------------------------   523
gb|CP006811.1_Lactobacillus_johnosnii_N62_936378_1872469                     -------------------------------------------------------------------------   523
dbj|AP014583.1_Winogradskyella_sp_PG2_2740238_1696625                        -------------------------------------------------------------------------   523
gb|CP007806.1_Brevibacillus_laterosporus_LMG15441_3876211_3876251            -------------------------------------------------------------------------   523
gb|CP007771.1_Campylobacter_lari_subsp_concheus_LMG11760_325484_1380655      -------------------------------------------------------------------------   523
gb|CP011074.1_Brevibacillus_laterosporus_2695574_2695534                     -------------------------------------------------------------------------   523
gb|CP012395.1_Clostridium_autoethanogenum_DSM10061_2383578_2414091           -------------------------------------------------------------------------   523
gb|CP013476.1_Turicibacter_sp_H121_1854744_1854792                           -------------------------------------------------------------------------   523
gb|CP015199.1_Chryseobacterium_glaciei_1481081_2039807                       -------------------------------------------------------------------------   523
gb|CP016400.1_Lactobacillus_johnsonii_257939_1274481                         -------------------------------------------------------------------------   523
emb|LT629794.1_Polaribacter_sp_Hel1_33_78_2572375_1908575                    -------------------------------------------------------------------------   523
gb|CP022346.1_Bacillus_thuringiensis_173658_173696                           -------------------------------------------------------------------------   523
gb|CP017705.1_Brevibacillus_laterosporus_DSM25_635195_635235                 -------------------------------------------------------------------------   523
gb|CP024731.1_Fusobacterium_periodonticum_950339_2375048                     -------------------------------------------------------------------------   523
gb|CP024700.1_Fusobacterium_periodonticum_2628426_1409491                    -------------------------------------------------------------------------   523
gb|CP024704.1_Fusobacterium_periodonticum_1050647_2200483                    -------------------------------------------------------------------------   523
gb|CP018259.1_Acinetobacter_bereziniae_667333_667376                         -------------------------------------------------------------------------   523
gb|CP025545.1_Brevibacillus_laterosporus_3840899_3840939                     -------------------------------------------------------------------------   523
gb|CP014218.1_Clostridium_botulinum_55907_55960                              -------------------------------------------------------------------------   523
gb|CP029736.1_Providencia_rettgeri_530944_530904                             -------------------------------------------------------------------------   523
gb|CP029614.1_Lactobacillus_johnsonii_354508_354461                          -------------------------------------------------------------------------   523
                                                                             .......460.......470.......480.......490.......500.......510.......520...
```

Pasteurella multocida PM70

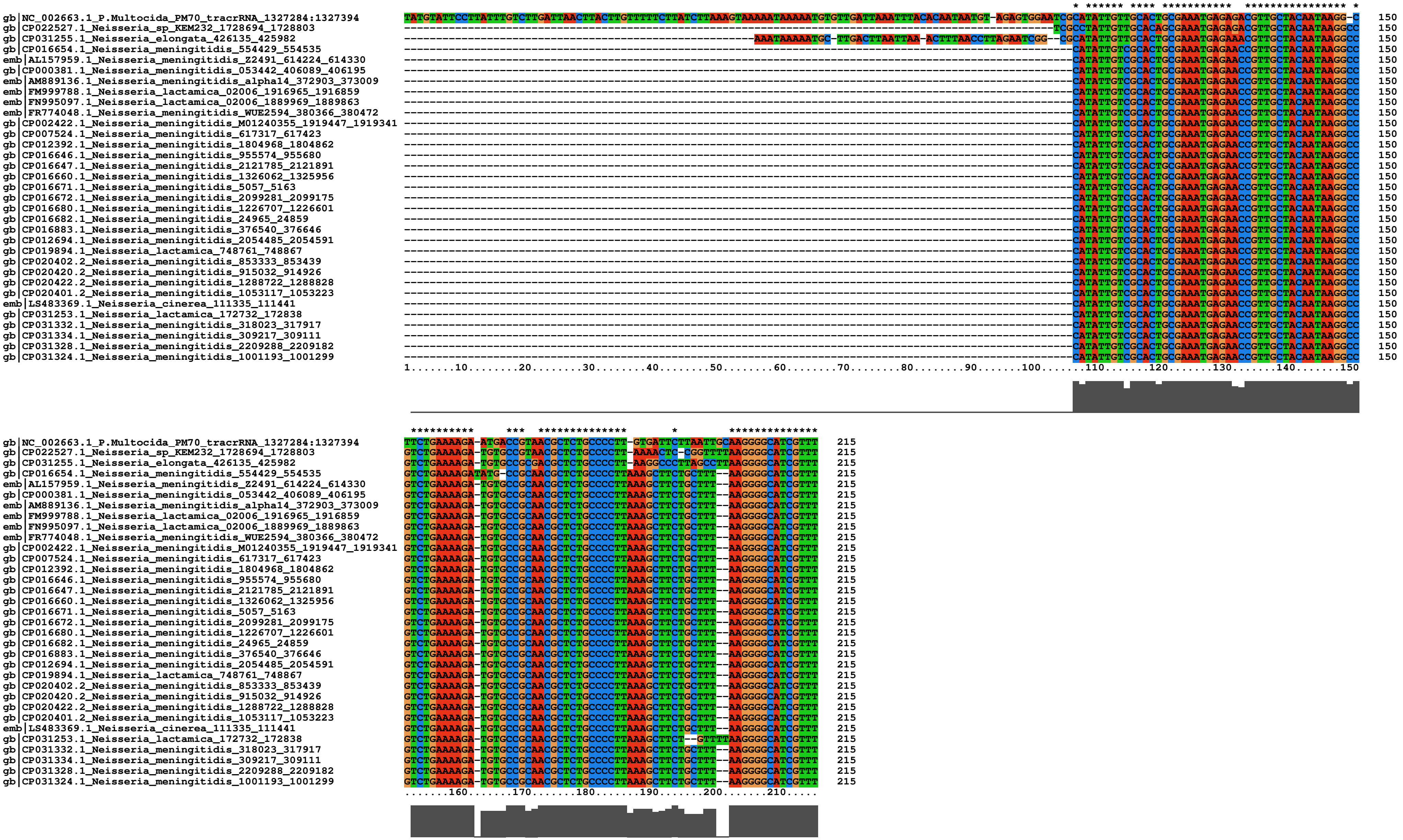

Haemophilus parainfluenzae T3T1

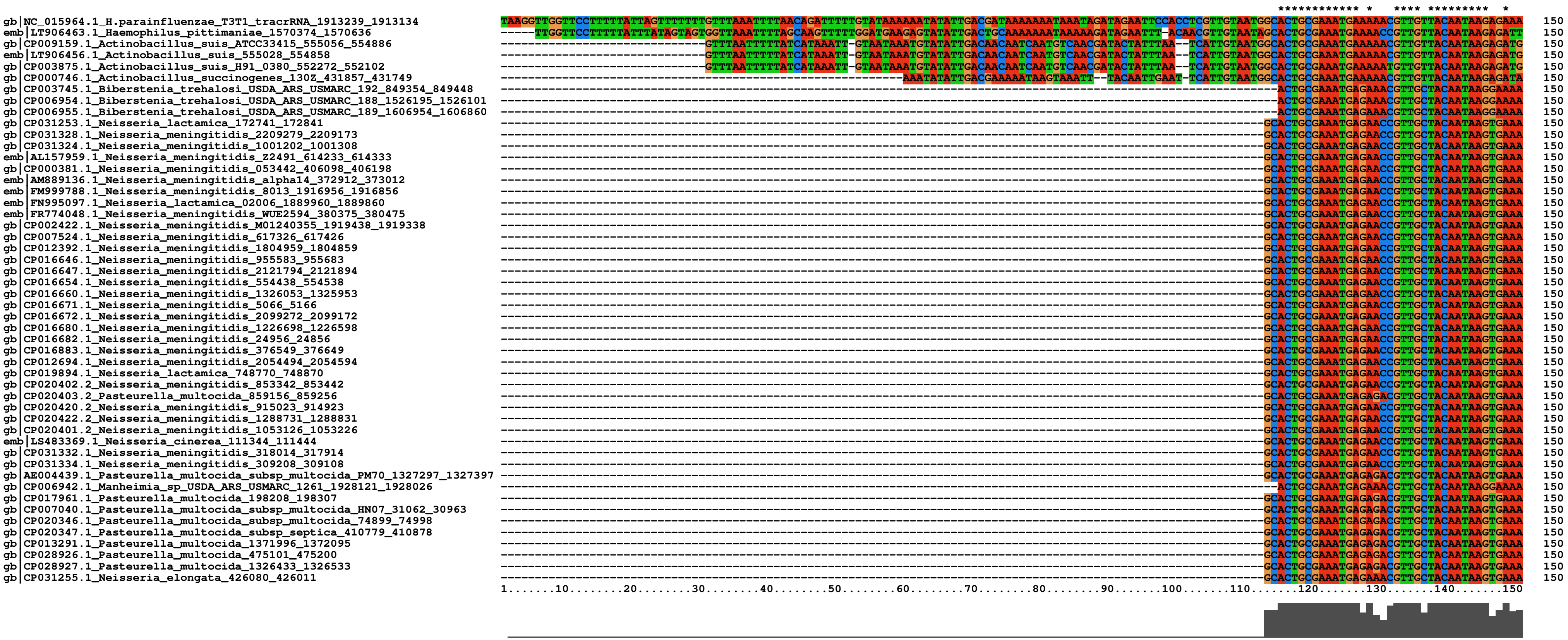

Haemophilus parainfluenzae T3T1

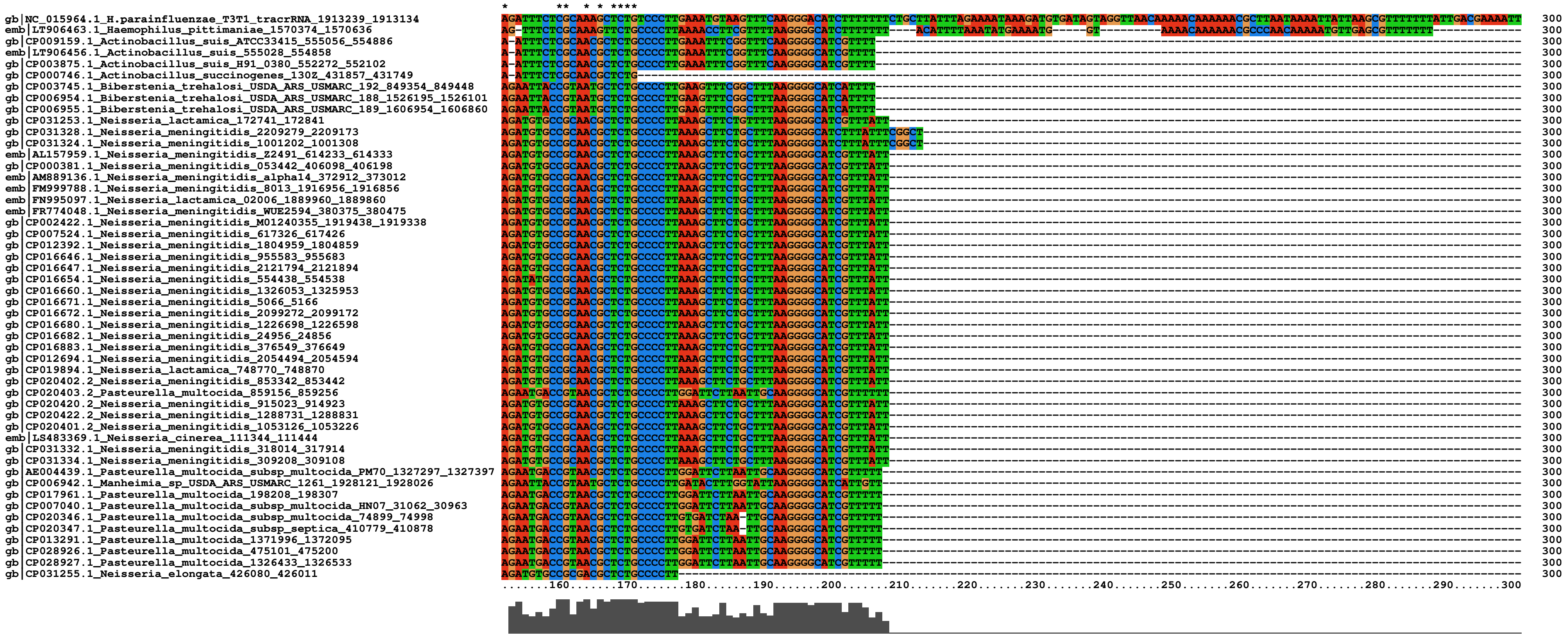

Haemophilus parainfluenzae T3T1

gb|NC_015964.1_H.parainfluenzae_T3T1_tracrRNA_1913239_1913134	TATA	304
emb|LT906463.1_Haemophilus_pittimaniae_1570374_1570636	----	304
gb|CP009159.1_Actinobacillus_suis_ATCC33415_555056_554886	----	304
emb|LT906456.1_Actinobacillus_suis_555028_554858	----	304
gb|CP003875.1_Actinobacillus_suis_H91_0380_552272_552102	----	304
gb|CP000746.1_Actinobacillus_succinogenes_130Z_431857_431749	----	304
gb|CP003745.1_Biberstenia_trehalosi_USDA_ARS_USMARC_192_849354_849448	----	304
gb|CP006954.1_Biberstenia_trehalosi_USDA_ARS_USMARC_188_1526195_1526101	----	304
gb|CP006955.1_Biberstenia_trehalosi_USDA_ARS_USMARC_189_1606954_1606860	----	304
gb|CP031253.1_Neisseria_lactamica_172741_172841	----	304
gb|CP031328.1_Neisseria_meningitidis_2209279_2209173	----	304
gb|CP031324.1_Neisseria_meningitidis_1001202_1001308	----	304
emb|AL157959.1_Neisseria_meningitidis_Z2491_614233_614333	----	304
gb|CP000381.1_Neisseria_meningitidis_053442_406098_406198	----	304
emb|AM889136.1_Neisseria_meningitidis_alpha14_372912_373012	----	304
emb|FM999788.1_Neisseria_meningitidis_8013_1916956_1916856	----	304
emb|FN995097.1_Neisseria_lactamica_02006_1889960_1889860	----	304
emb|FR774048.1_Neisseria_meningitidis_WUE2594_380375_380475	----	304
gb|CP002422.1_Neisseria_meningitidis_M01240355_1919438_1919338	----	304
gb|CP007524.1_Neisseria_meningitidis_617326_617426	----	304
gb|CP012392.1_Neisseria_meningitidis_1804959_1804859	----	304
gb|CP016646.1_Neisseria_meningitidis_955583_955683	----	304
gb|CP016647.1_Neisseria_meningitidis_2121794_2121894	----	304
gb|CP016654.1_Neisseria_meningitidis_554438_554538	----	304
gb|CP016660.1_Neisseria_meningitidis_1326053_1325953	----	304
gb|CP016671.1_Neisseria_meningitidis_5066_5166	----	304
gb|CP016672.1_Neisseria_meningitidis_2099272_2099172	----	304
gb|CP016680.1_Neisseria_meningitidis_1226698_1226598	----	304
gb|CP016682.1_Neisseria_meningitidis_24956_24856	----	304
gb|CP016883.1_Neisseria_meningitidis_376549_376649	----	304
gb|CP012694.1_Neisseria_meningitidis_2054494_2054594	----	304
gb|CP019894.1_Neisseria_lactamica_748770_748870	----	304
gb|CP020402.2_Neisseria_meningitidis_853342_853442	----	304
gb|CP020403.2_Pasteurella_multocida_859156_859256	----	304
gb|CP020420.2_Neisseria_meningitidis_915023_914923	----	304
gb|CP020422.2_Neisseria_meningitidis_1288731_1288831	----	304
gb|CP020401.2_Neisseria_meningitidis_1053126_1053226	----	304
emb|LS483369.1_Neisseria_cinerea_111344_111444	----	304
gb|CP031332.1_Neisseria_meningitidis_318014_317914	----	304
gb|CP031334.1_Neisseria_meningitidis_309208_309108	----	304
gb|AE004439.1_Pasteurella_multocida_subsp_multocida_PM70_1327297_1327397	----	304
gb|CP006942.1_Manheimia_sp_USDA_ARS_USMARC_1261_1928121_1928026	----	304
gb|CP017961.1_Pasteurella_multocida_198208_198307	----	304
gb|CP007040.1_Pasteurella_multocida_subsp_multocida_HN07_31062_30963	----	304
gb|CP020346.1_Pasteurella_multocida_subsp_multocida_74899_74998	----	304
gb|CP020347.1_Pasteurella_multocida_subsp_septica_410779_410878	----	304
gb|CP013291.1_Pasteurella_multocida_1371996_1372095	----	304
gb|CP028926.1_Pasteurella_multocida_475101_475200	----	304
gb|CP028927.1_Pasteurella_multocida_1326433_1326533	----	304
gb|CP031255.1_Neisseria_elongata_426080_426011	----	304
	....	

Neisseria meningitidis ATCC 13091

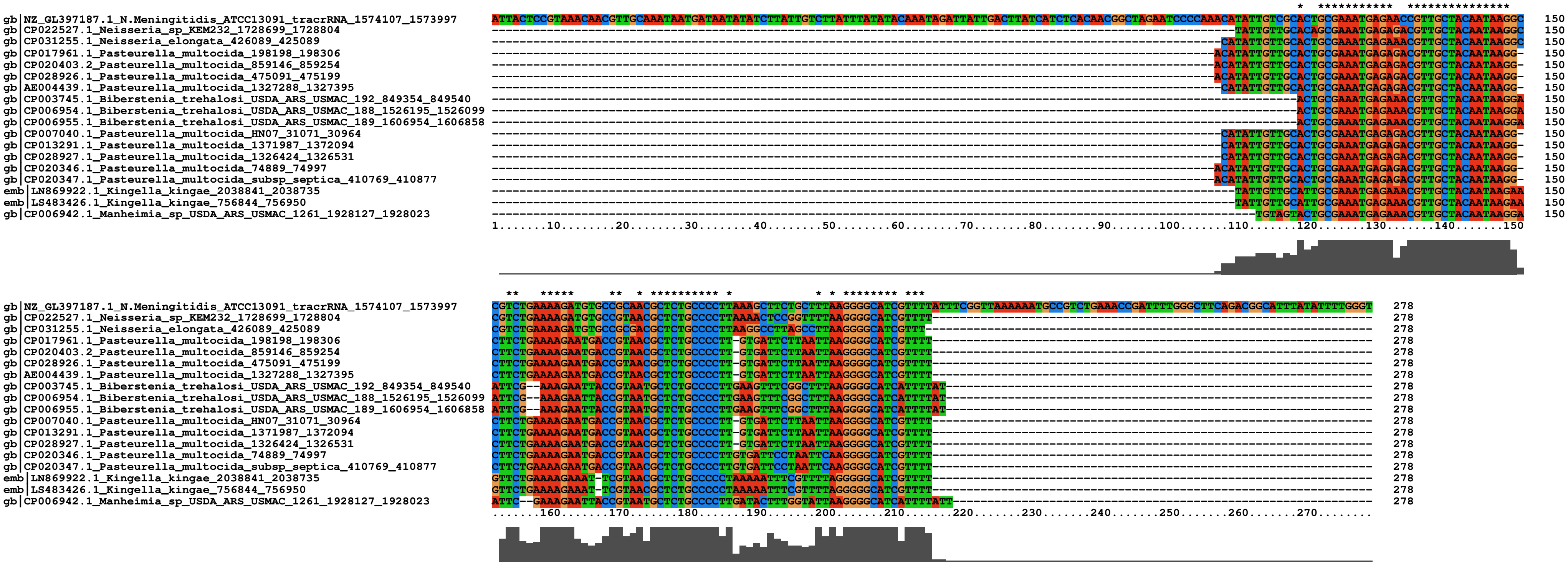

Neisseria lactamica 020 06

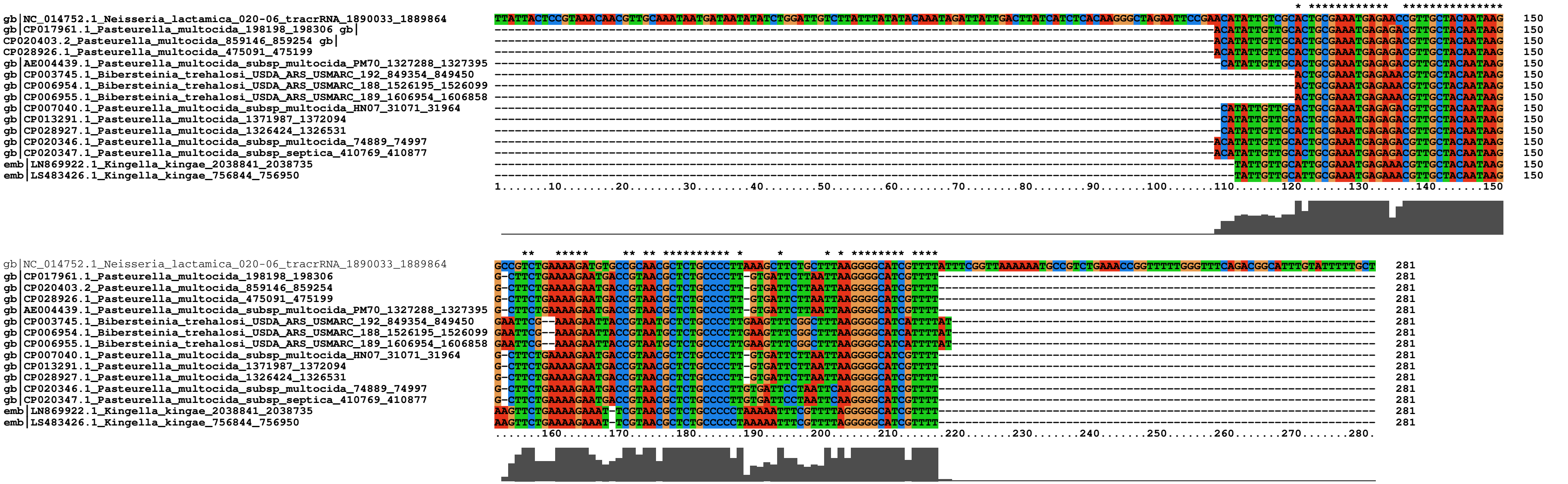

Listeria innocua CLIP1262

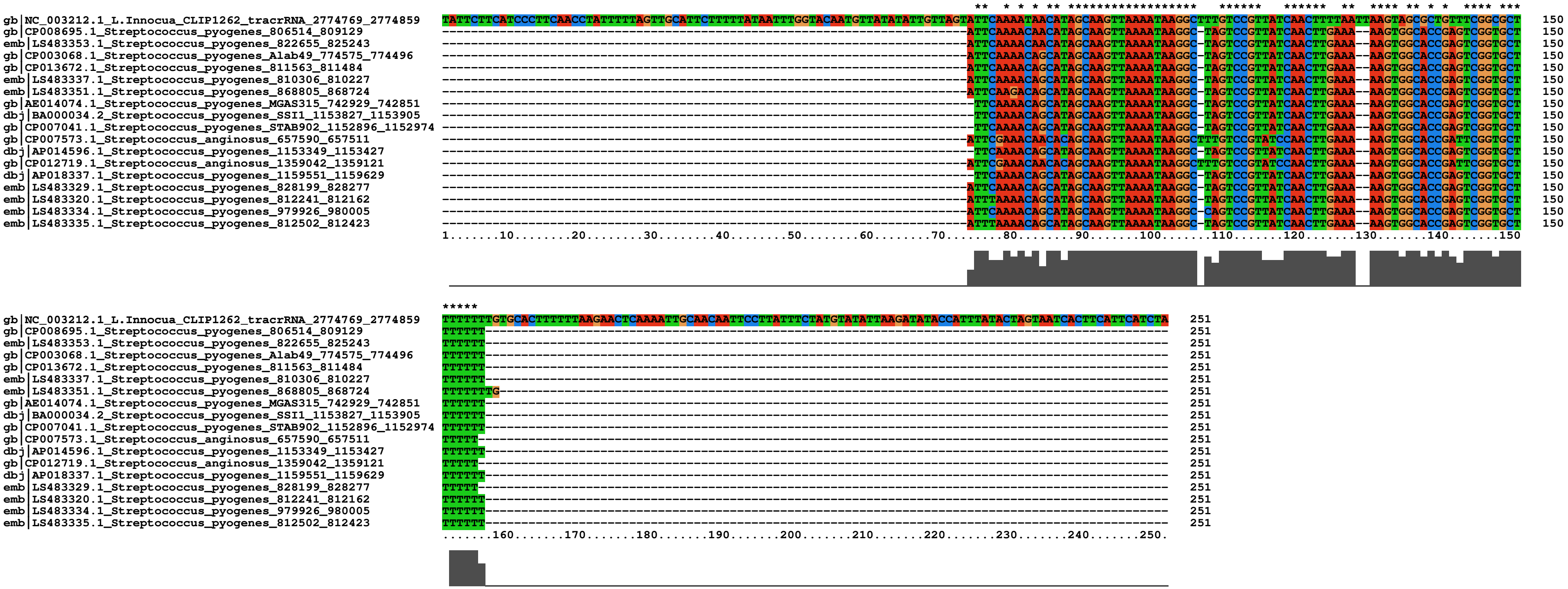

Listeria monocytogenes SLCC2482

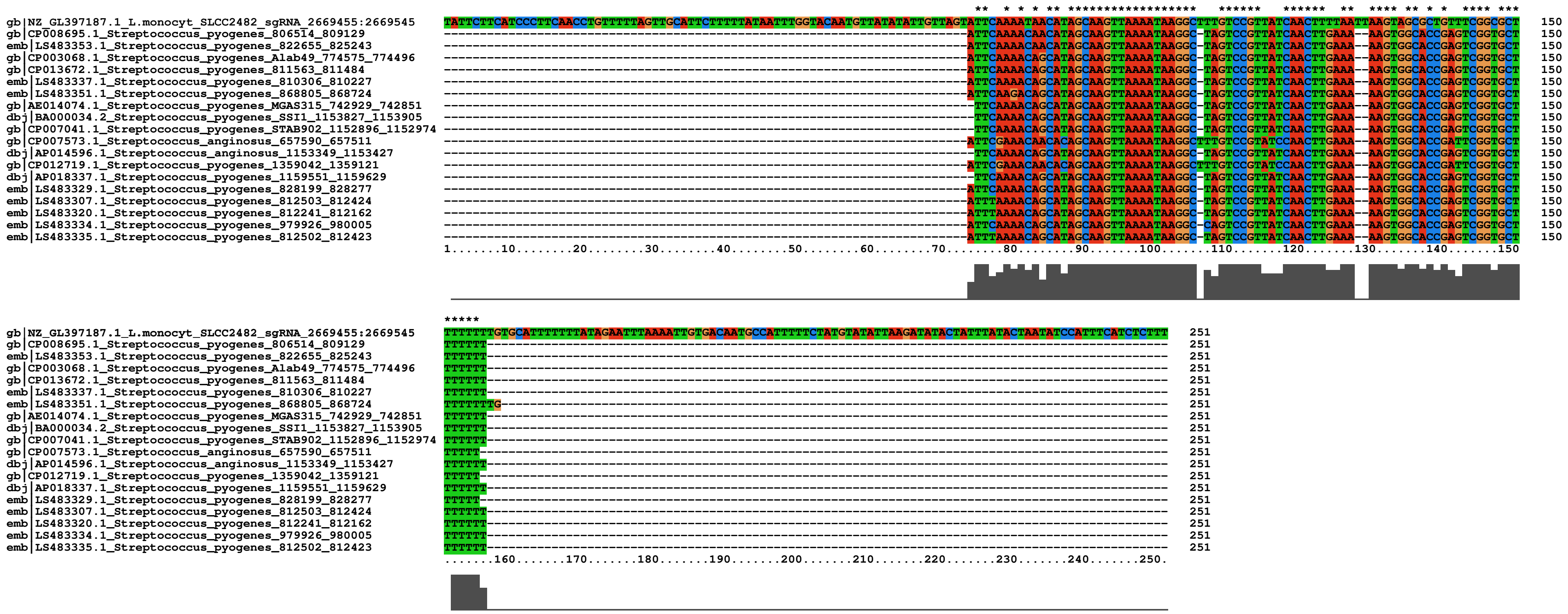

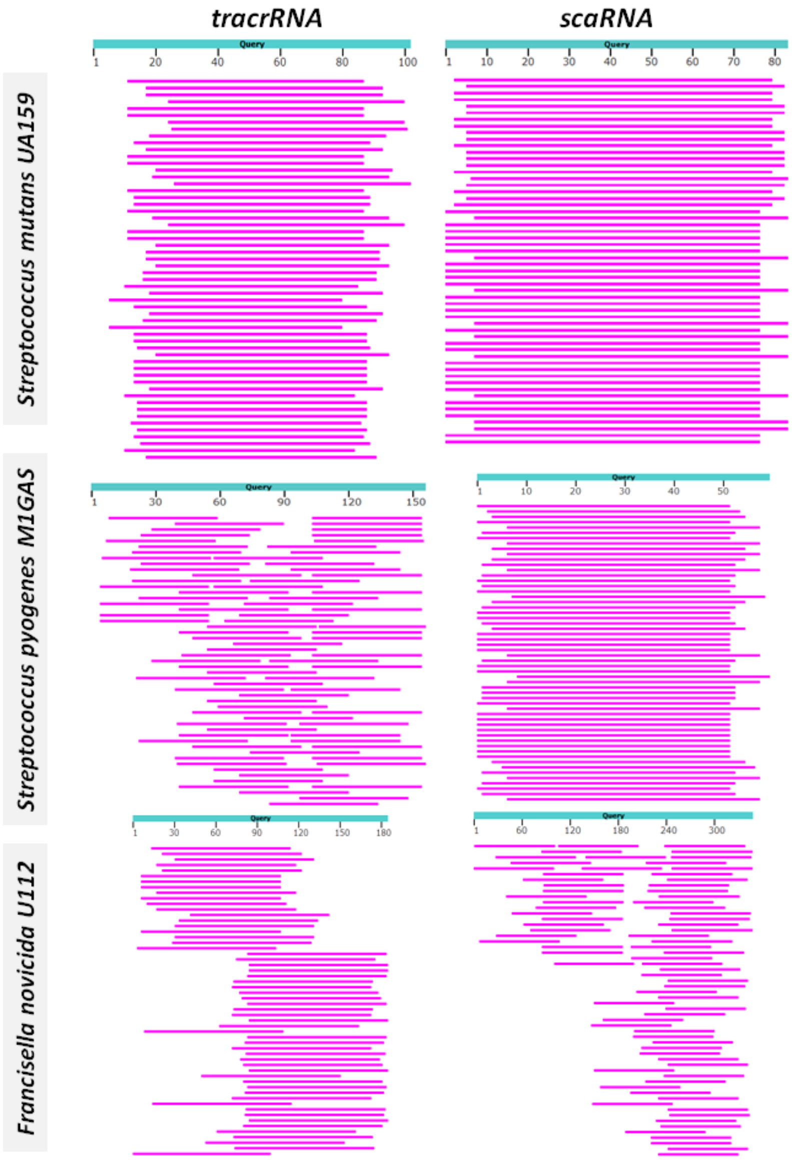

Supplementary Figure S2. Matches of RNA-Seq reads to tracrRNA and scaRNA predictions in *Streptococcus mutans UA159*, *Streptococcus pyogenes M1GAS* and *Francisella novicida U112*. In the panel, the alignment graphical summary, for tracrRNA (left column), and scaRNA (right column) predictions against SRA database for a given strain is indicated. For *S. mutans UA159* scaRNA prediction short upstream flanking sequence was included to indicate the alignment against entire RNA-Seq reads when possible.

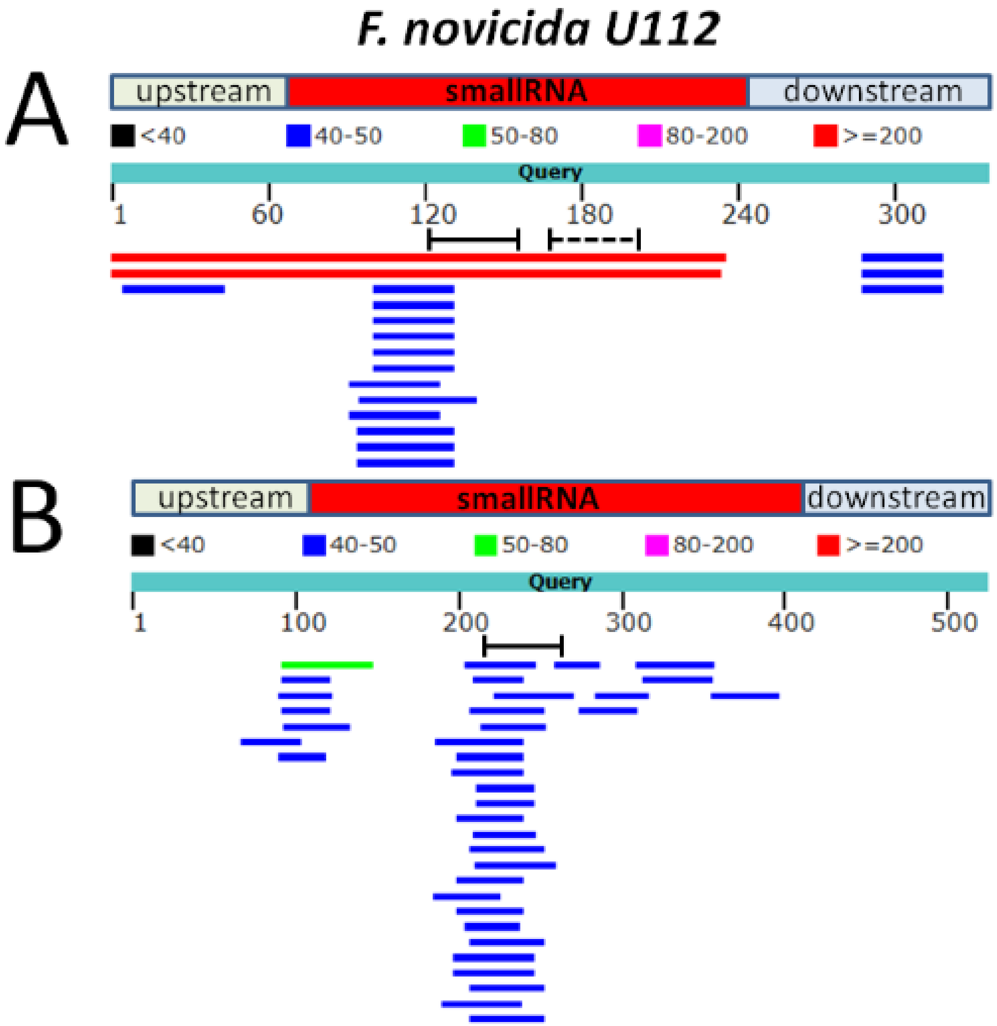

Supplementary Figure S3. Conservation of tracrRNA and scaRNA with flanking regions in *Francisella novicida U112*. In the upper panel (A), conservation of tracrRNA along with the upstream and downstream flank is shown as alignment graphical summary; length of each segment is indicated by the corresponding rectangle (light green for upstream flank, red for RNA, and light blue for downstream flank) with coordinate axis indicated; scaRNA-pairing region is indicated by the solid black, while the target-recognition region by the dashed black horizontal bar; In the lower panel (B), the same information is provided for scaRNA, where solid black horizontal bar now indicates tracrRNA-pairing region. Note that it is tracrRNA, which recognizes mRNA target.

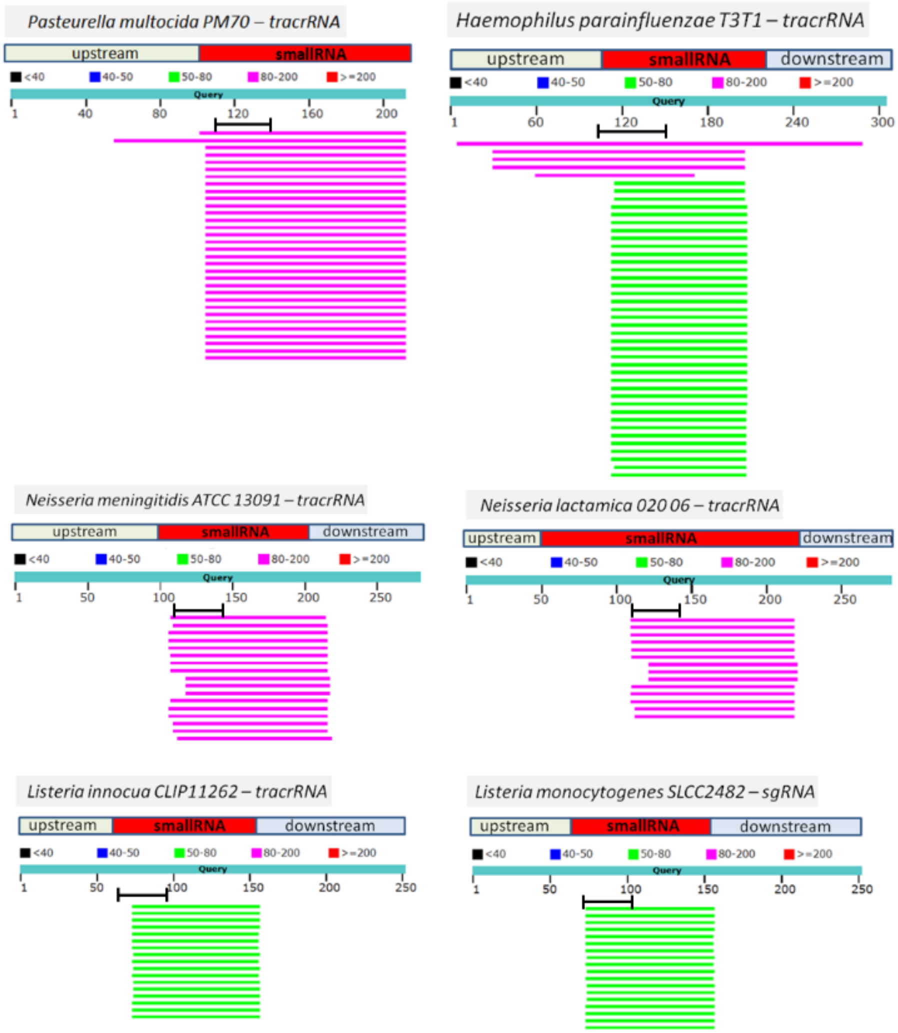

Supplementary Figure S4. Conservation for more deeply conserved predicted small RNAs. Conservation for the predicted CRISPR-associated small RNAs (conserved at class or phylum level), together with their flanking regions, is shown as alignment graphical summary; for each small RNA the strain of origin is indicated above the alignment, the upstream flank, small RNA and downstream flank segments are indicated by the corresponding green, red and blue rectangles (see caption for Figure S3), while the region of homology with the CRISPR array is indicated by the solid black horizontal bar.
